# Genome assembly and protein structure modeling reveal key molecular features of divergent *wmk* homologs in *Wolbachia*

**DOI:** 10.1128/spectrum.02893-25

**Published:** 2025-12-30

**Authors:** Ranjit Kumar Sahoo, Naveen Kumar Chandrakumaran, Karthikeyan Vasudevan

**Affiliations:** 1 CSIR - Centre for Cellular and Molecular Biology 28569, Hyderabad, India; Brigham Young University, Provo, Utah, USA

**Keywords:** AlphaFold, host-symbiont interaction, phage WO, male killing, helix-turn-helix, *Zygogramma bicolorata*

## Abstract

*Wolbachia* induces female-biased sex ratios in host populations through male-specific killing, thereby enhancing its spread via maternal transmission. The prophage-associated gene *wmk* has been proposed as a key effector underlying this male-killing (MK) phenotype. Interestingly, *wmk* homologs are found across diverse *Wolbachia* strains, regardless of the presence of male killing, and show extensive sequence divergence. However, the functional implications of this sequence variation—particularly among distant homologs—remain poorly understood. Here, we analyzed *wmk* homologs from 18 *Wolbachia* genomes, including 17 publicly available genomes and one de novo assembled genome from the parthenium beetle. We identified a highly divergent *wmk* homolog in the latter and predicted its protein structure using AlphaFold2, followed by molecular dynamics simulations to characterize its molecular features. Our analyses reveal that *wmk*-encoded protein exhibits a modular architecture comprising two helix-turn-helix (HTH) domains and an additional accessory domain not previously described. Modeling of inter-domain interactions further supports the functional relevance of this modular organization. Comparative analyses across all homologs distinguish divergent *wmk* from other canonical variants based on sequence composition, structural organization, and phylogenetic clustering. Notably, the structural features that differentiate these homologs also distinguish *wmk* from other known HTH-type regulators. Together, our findings provide new molecular insights into the architecture and evolution of *wmk*, offering a framework to understand the mechanistic basis of the MK phenotype in *Wolbachia*.

**IMPORTANCE:**

*Wolbachia*-induced male killing presents a promising strategy for the biocontrol of vector and pest populations. The *wmk* gene has been identified as a candidate underlying this phenotype. Yet, the significance of its sequence variation—particularly between highly divergent homologs—remains unclear. Here, we characterize a divergent *wmk* homolog from a novel *Wolbachia* strain. Then, we compare sequence and structural features of *wmk* homologs across a total of 18 *Wolbachia* strains using AlphaFold2 and molecular dynamics simulations. Our results highlight key molecular features in divergent variants and provide new insights into *wmk* evolution, laying a basis for exploring its functional diversity across *Wolbachia* lineages.

## INTRODUCTION

Endosymbionts of arthropod hosts, particularly secondary symbionts, have evolved diverse strategies to persist within host populations (1). These symbionts exhibit a wide ecological spectrum with their hosts, ranging from beneficial associations (2–5) to modification of reproduction (6, 7). The net outcome of these interactions is often environment-dependent, leading to variation in symbiont prevalence across host populations. For example, in the absence of parasitism by wasps, the frequency of *Hamiltonella*-infected pea aphids—otherwise protected from the parasitism—declines in experimental populations (8). Symbionts that modify host reproduction achieve high prevalence by conferring reproductive advantages to infected female hosts over uninfected ones, or by inducing female-biased sex ratios, or potentially through a combination of both mechanisms. As a result, secondary symbionts profoundly influence the population dynamics and genetic structure of their hosts (9–11).

Maternally transmitted endosymbionts of the genus *Wolbachia* (gram-negative, alpha-proteobacterium) infect ~52% of arthropod species worldwide (12). Beyond beneficial effects (reviewed in reference [13]), *Wolbachia* manipulates host reproduction through mechanisms such as cytoplasmic incompatibility (CI) between infected males and uninfected females, male-to-female feminization, parthenogenesis induction, or selective male killing (MK) (13, 14). Except for CI, all of these mechanisms bias host sex ratios in favor of infected females, thereby promoting bacterial transmission. Such distortion can lead to extreme sex ratio imbalances in host populations, as observed in the butterfly *Hypolimnas bolina*, for example, where infection with a MK strain resulted in female-biased sex ratios as high as 100:1 (reviewed in [15]). This interaction has motivated the proposed joint application of MK and the sterile-insect technique (SIT) as a new strategy for vector or pest control (16, 17).

MK in *Wolbachia* was first observed in the beetle *Adalia bipunctuata* and the butterfly *Acraea encedon*, wherein the male progeny of the infected female undergoes developmental arrest during early embryogenesis (18). Later investigations in *Drosophila bifasciata* revealed that the bacterium induces embryonic DNA damage by interfering with dosage compensation (DC) in male hosts (19–22). DC is an epigenetic process to equalize expression levels of sex-linked genes between male and female (23) and has been a molecular target for reproduction modification by bacterial symbionts (24). Toward the genetic basis of MK, the role of the *oscar* gene in *Wolbachia* has recently been elucidated (25), where the *oscar*-encoded protein reduces Masc protein accumulation—an essential component in masculinization and DC in males (26). However, the *oscar* gene has only been reported in *Wolbachia* strains infecting Lepidoptera (15, 25, 27–29), suggesting that its function may be tightly linked to the unique sex-determining pathway or DC mechanism of this group.

In contrast, *wmk* (WO-mediated killing) has been proposed as a strong candidate gene for *Wolbachia* MK effect (17), and its homologs are found in *Wolbachia* infecting several insect orders including Lepidoptera (15, 17, 30). Due to its broader taxonomic distribution, *wmk* is expected to function through a distinct molecular mechanism compared to *oscar*. Although the exact mechanism remains unknown, *wmk* is predicted to act as a DNA-binding transcriptional regulator (17). Transgenic expression of codon-optimized *wmk* from the *Wolbachia* strain *w*Mel (host: *Drosophila melanogaster*) has revealed early embryonic DNA defects in *D. melanogaster* (17), similar to those seen in natural infection of the MK strain *w*Bif in *D. bifasciata* (20–22).

Unlike the well-established relationship between the *oscar* gene and the MK phenotype (15, 25, 27–29), the *wmk* gene is not consistently associated with this phenotype (17). For example, *wmk*-carrying *w*Mel strain is not known to induce MK in its natural occurrence within *D. melanogaster*. Yet, transgenic expression of the gene reveals its MK effect (17), strongly suggesting the role of host genotype and environmental factors on phenotype expression. Furthermore, *wmk* homologs are present in both MK and non-MK *Wolbachia* strains. It is possible that *wmk* in non-MK strains represents a transient state in an ongoing evolutionary arms race between the bacterium and its hosts. Perlmutter et al. (31) explored this complex genotype-phenotype relationship in *w*Mel and demonstrated that even a single synonymous codon change in *wmk*, particularly toward the initial part of the sequence, can alter the severity of MK within the same host genetic background. Therefore, not all synonymous mutations in *wmk* may conform to their functional redundancy (31).

The variation in the *wmk* sequence may also be explained by the sensitivity of amino acid substitution to host cellular and molecular processes and sex-determination systems (17). An evolutionary arms race between *Wolbachia* and its hosts could further drive sequence optimization of *wmk*. Within this framework, a high degree of *wmk* divergence is expected, given the diversity of sex-determination systems and biochemical processes among arthropods. Consistent with this model, a *wmk* variant identified in the MK strain *w*Bif exhibited only 26% amino acid similarity to the reference *w*Mel-*wmk* (17). This low sequence identity was striking, as *wmk* from other MK *Wolbachia* strains typically exhibit higher identity to *w*Mel-*wmk*, ranging from 72.9% to 99.6% (BlastP-based) (17). Despite this sequence divergence between *w*Bif-*wmk* and *w*Mel-*wmk*, predicted protein models (using Phyre2) suggested their structural redundancy (17).

Taken together, previous studies suggest that while modest sequence variation in *wmk* can have functional consequences, extensive divergence may still preserve a degree of functional redundancy through structural conservation. Although this possibility cannot be excluded, we sought to reassess it in the light of the discovery of another divergent *wmk* variant. We investigate whether extensive sequence divergence between *wmk* variants preserves structural redundancy. Our study design incorporates the key molecular features of *wmk*-encoded protein into the molecular divergence analyses. The newly identified *wmk* variant was discovered in a *Wolbachia* strain from the beetle *Zygogramma bicolorata* (Coleoptera: Chrysomelidae), a widely used biocontrol agent against the invasive weed *Parthenium hysterophorus* (32). Protein structures of *wmk* variants, comprising the newly identified sequence as well as previously reported ones from 17 *Wolbachia* strains, were inferred using state-of-the-art methods: AlphaFold2 (AF2) for structure prediction (33) and GROMACS for molecular dynamics (MD) simulation (34). Our findings reveal the unique molecular architecture of the *wmk*-encoded protein and highlight key structural features that distinguish *w*Bif-Wmk and the newly identified variant from *w*Mel-Wmk and its closely related homologs.

## RESULTS

### Phage-associated *wmk* homolog in *w*Zbi

**Fig 1:**
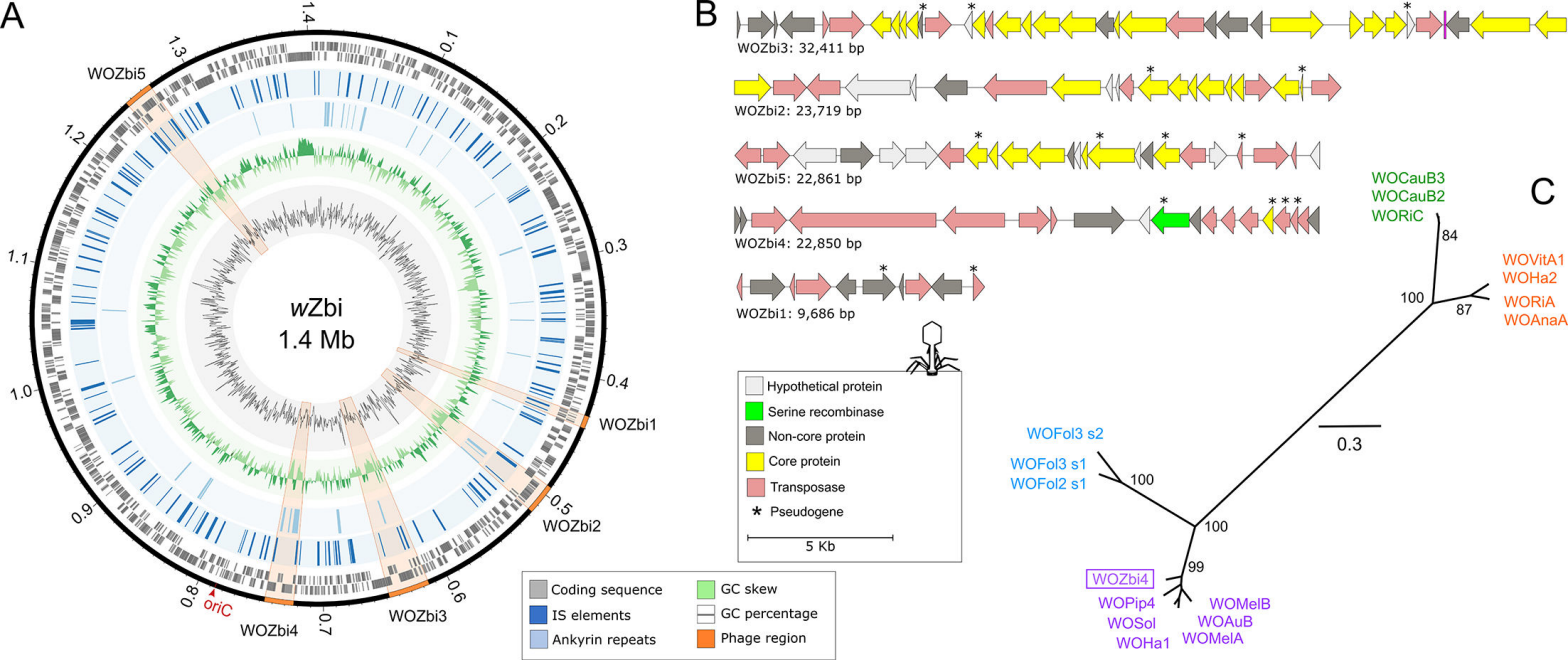
(**A**) Circos plot of *w*Zbi genome with the inner tracks representing, from circumference to center, the coding sequence regions, insertion sequence elements, ankyrin repeats, GC skew, and GC percentage (range: 0.27–0.49). GC skew and GC percentage were calculated with 1,000 bp window size and step size. Phage regions are highlighted in orange color shades. (**B**) Molecular feature of the phage regions showing gene annotation and size of the regions. (**C**) Maximum likelihood tree of serine recombinase gene retrieved from 17 WO phage elements. Rectangular box indicates sequence from *w*Zbi. WO phages are color-coded as per their known classification: green-sr1WO, orange-sr2WO, violet-sr3WO, and blue-sr4WO. Node values indicate nodal support from 1,000 bootstrap replicates.

Using 476 Mb of *Wolbachia*-specific long-reads retrieved from whole-genome sequencing of the host beetle *Z. bicolorata* (see reference [32]), we assembled a 1,408,150 bp complete genome of a novel *Wolbachia* endosymbiont, named *w*Zbi (Fig. 1A). To minimize environmental microbial contamination, freshly eclosed female adult beetle—largely devoid of larval gut contents—was deprived of food for 48 h post-eclosion and surface-sterilized with ethanol, as described in Sahoo et al. (32). *w*Zbi assembly quality matrices indicated high completeness (BUSCO: 99.1% for both genome and proteins; checkM: 99.8%) and low contamination (checkM: 0.75%) (Table S1). Assembly graphs confirmed the circular nature of the genome, and additionally, we identified the variable region of replication origin (oriC) within the genome (Fig. 1A, Fig. S1). Read depth calculation indicated ~300× genome-wide coverage (Fig. S1). We could not find evidence for the occurrence of a plasmid in *w*Zbi. Phylogenomic analysis of 242 *Wolbachia* genomes (using 350 shared BUSCO gene markers) and GC-content analysis of 126 complete genomes placed *w*Zbi within supergroup-A clade (Fig. S2). Despite showing ~97% ANI and >94% shared orthologs with its closely related lineages, *w*Zbi displayed extensive genome rearrangements (Fig. S3). Sequence typing against the PubMLST-*Wolbachia* database indicated novel alleles at four out of five loci in multi-locus sequence typing and one out of four loci in WSP-based typing (Table S2).

Notably, we identified five prophage regions in *w*Zbi, named WOZbi1 through WOZbi5, ranging in size from 9.6 to 32.4 kb and collectively comprising 111.5 kb, approximately 10% of the complete genome (Fig. 1B). These WOZbi elements included a large segment (*ca*. 36 kb, or one-third) of ankyrin repeat-containing genes and transposases, primarily from the IS66 and IS630 families. Of the estimated 110 gene regions encoded within the WOZbi elements, approximately 15 appeared to be pseudogenized and occurred across all phage elements (Fig. 1B). The *w*Zbi serine recombinase, a key marker for WO phage classification (35), grouped within the sr3WO clade in our phylogenetic analysis (Fig. 1C), suggesting that WOZbi1-5 regions—particularly WOZbi4—likely originated from an ancestral sr3WO phage infection. However, we cannot exclude the possibility that additional ancestral WO phage infections went undetected, either due to recombinase gene degradation or complete loss of this marker in the ancestral lineages.

**Fig 2:**
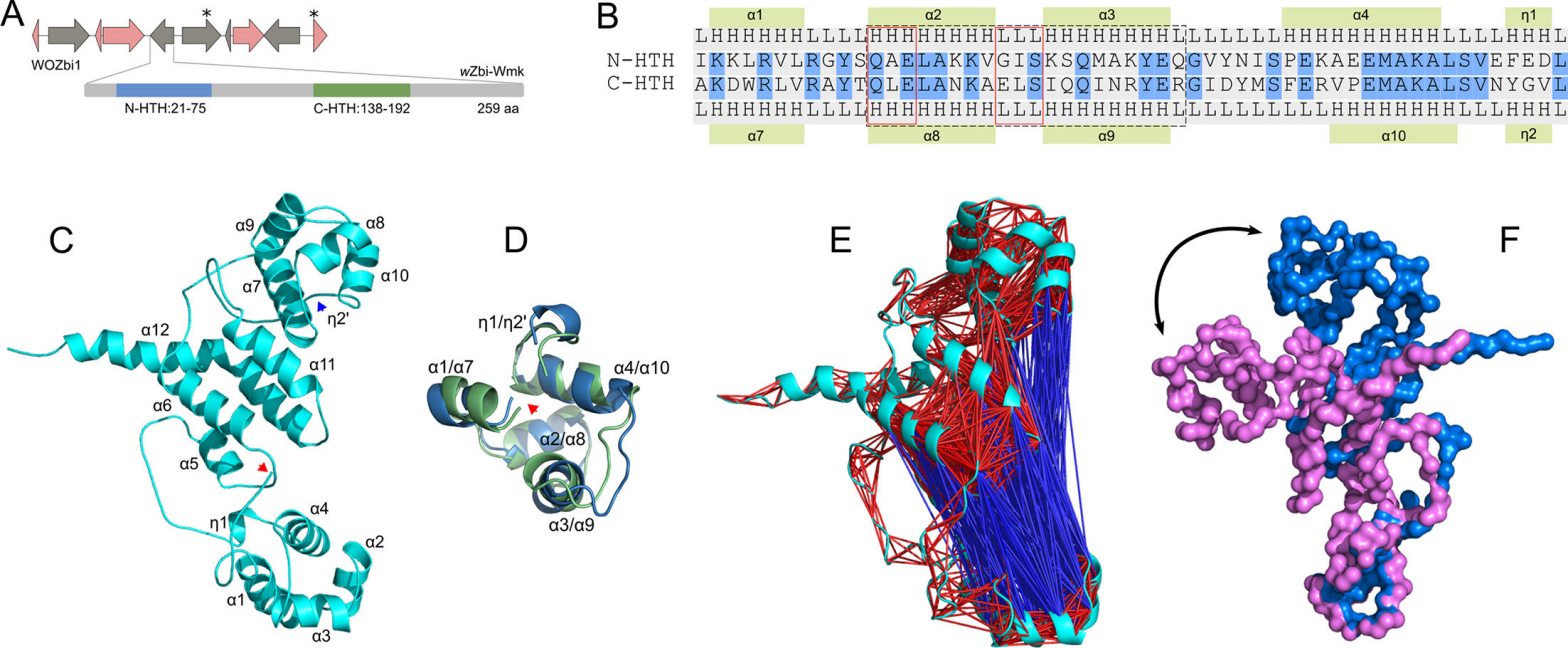
(**A**) Molecular feature of *w*Zbi-Wmk highlighting N- and C-terminal HTH domains. The domains are ranged between 21–75 and 138–192 residues, respectively, and the protein is 259 residues long. The corresponding gene *w*Zbi-*wmk* lies within the phage element WOZbi1, which is described in Fig. 1B. (**B**) Residues in N-HTH and C-HTH domains are aligned. Blue color highlights the matching residues. Secondary folds (H: helix; L: loop) and associated alpha helices (α1-α4, α7-α10, ƞ1, and ƞ2) from the AlphaFold2-predicted structure are mentioned beside the residues. The red-boxed regions indicate the conserved “phs” (left) and “shs” (right) patterns, while the dashed box denotes the predicted DNA-binding regions. (**C**) The representative conformation of *w*Zbi-Wmk was sampled at 85 ns time point from the MD simulation of AlphaFold2-predicted model. Red arrow mark indicates N-terminal. While α1-α12 indicate complete alpha helices, ƞ1 represents incomplete helix, possibly a 310-type. The blue mark suggests the absence of ƞ2 (denoted as ƞ2′) from the conformation state (compared to the AF2-predicted model in Fig. S6). (**D**) Structural alignment of N-HTH (blue) and C-HTH (green) domains from the representative *w*Zbi-Wmk conformation. Overlapped alpha helices of the domains are labeled. Red arrow indicates N-terminal. (**E**) Results from cross-correlation analysis indicating both positively (red) and negatively (blue) correlated conformational motions for *w*Zbi-Wmk. See Fig. S9 for independent visualization of these motions. (**F**) Conformations showing the extent of motion captured in Principal Component 1 from the principal component analysis (detail in Fig. S10).

Our investigation into *Wolbachia* genes known to manipulate host reproduction revealed that homologs of *piff* (parthenogenesis inducing factor) and *cifA* (A-component of CI-inducing factor) were absent in *w*Zbi. We also found no clear evidence for *cifB* (B-component of CI-inducing factor) or *oscar* (a male-killing [MK] gene) (see Fig. S4). Interestingly, three gene regions in *w*Zbi (CP149530: WKH12_00075, WKH12_02010, and WKH12_05225) were initially identified as homologs of *wmk* based on sequence similarity to reference *wmk* genes from multiple *Wolbachia* strains (see Materials and Methods). However, manual curation and reciprocal best-hit homology searches ultimately supported only one of these regions as a putative *wmk* gene. This candidate gene, *w*Zbi-*wmk* (WKH12_02010), encodes a 259-amino-acid protein located within the phage region WOZbi1 (Fig. 2A). WOZbi1 appears to be an Eukaryotic Association Module (EAM) or a degenerated remnant of an ancestral EAM, as it lacks core phage structural genes and contains several mobile elements along with a pseudogenized *ligA* gene. The other two homologs (WKH12_00075 and WKH12_05225) were shorter in length (120 and 101 amino acids, respectively), less than half the size of *w*Zbi-*wmk*, distantly similar to *w*Zbi-*wmk* (blastp: <30% identity), and occurred outside the phage regions (details in Fig. S5). The significance of these two homologs relative to *w*Zbi-*wmk* may provide useful insights into *wmk* gene evolution.

### *w*Zbi-Wmk displays modular architecture

To gain structural and functional insights into *w*Zbi-*wmk*, we annotated the sequence for conserved domains and predicted the protein structure using AlphaFold2 (AF2) (Fig. S6) (33). We then performed MD simulations on the AF2-predicted structure to account for the dynamic nature of the protein and to validate key molecular features (34, 36). While the overall structural characteristics remained largely unchanged, except for a few minor transitions as described below, the protein adopted a more closed configuration during the simulation. This shift led to a marked increase in the root-mean-square deviation (RMSD) between the equilibrated MD models and the original AF2 prediction (Fig.S7). To capture conformational variability, we sampled eight evenly spaced models from the MD trajectory (between 25 and 95 nanoseconds [ns] with a 10 ns interval) for using them in comparative analyses described in later sections and randomly selected one of them (recovered at 85 ns) as the representative structural model of *w*Zbi-Wmk.

Our evaluation revealed that *w*Zbi-Wmk exhibits a modular architecture comprising two structured domains, an inter-domain linker (IDL), and N- and C-terminal extensions. Consistent with the overall protein architecture observed in *w*Mel-Wmk and its homologs (17), *w*Zbi-Wmk contains two helix-turn-helix (HTH) domains of the cro/C1-type (Fig. 2A; Table S3). The N-terminal HTH (N-HTH) spans residues 21–75 (score: 16.046), while the C-terminal HTH (C-HTH) spans residues 138–192 (score: 10.994), separated by a 62-residue IDL (ScanProSite prediction [37, 38]). Although the two domains share only ~45% sequence identity, both adopt a similar four-helix bundle configuration (N-HTH: α1-α4, C-HTH: α7-α10) (Fig. 2B and D), with structural concordance (RMSD =2.145 Å over 55 residues). Both domains also contain a partial helix (ƞ1, ƞ2), likely 310-helix, toward the C-terminus, although MD simulation suggests this feature may represent a transient conformation (Fig. 2C and D; Fig. S6).

We identified two residue patterns that are conserved among HTH domains across all kingdoms of life (39): (i) a “shs” (small-hydrophobic-small) pattern between second and third helices within each domain (N-HTH: GIS; C-HTH: ELS), and (ii) a “phs” (polar-hydrophobic-small) pattern in second helix of each domain (N-HTH: QAE; C-HTH: QLE) (Fig. 2B). This widely conserved sequence elements, particularly the hydrophobic residues, are part of the hydrophobic core that stabilizes the domain (39). This highlights that the two middle helices of each *w*Zbi-Wmk domain (N-HTH: α2-α3; C-HTH: α8-α9) likely represent the core DNA-binding motif, which exactly matches with the predicted DNA-binding regions spanning residues 32–51 and 149–168, respectively (Fig. 2B). The overall positive surface charge around these motifs supports their putative role in nucleic acid binding (Fig. S8). Finally, a structure homology search of the domains using DALI (40) identified the five-helical domain of the phage protein ImmR from *Bacillus subtilis* (PDB: 7T8I) as the closest match (DALI: z >9.6), supporting the predicted function of *w*Zbi-Wmk as a transcriptional regulator.

Additionally, *w*Zbi-Wmk contains a relatively long IDL comprising 62 residues arranged in two α-helices and three loops. This architecture likely facilitates domain-domain mobility as observed in the sampled conformations from the MD simulation (Fig. S8). We hypothesize that the linker may serve as a flexible hinge enabling dynamic motion between the domains (Video S1). We investigated these conformational dynamics by subjecting the MD trajectory of *w*Zbi-Wmk to cross-correlational analysis (CCA) and principal component analysis (PCA). CCA assessed correlated motions between structural elements across equilibrated conformations, while PCA captured the principal directions of variance and the contribution of individual conformations to overall system motion. Results from CCA revealed negatively correlated motions between the two HTH domains, suggesting their relatively opposite and hinge-like motions with respect to one another (Fig. 2E; Fig. S9). From PCA (Fig. S10), inspection of structures represented by the principal component 1 (PC1), which describes 58.4% of total variance, illustrated that the two HTH domains exhibit anti-correlated motion, transitioning between open and closed configurations. These transitions likely involve interactions between the IDL and N-HTH domain, causing the C-HTH domain to bend toward the N-HTH in the closed conformation (Fig. 2F). Structures for PC2, which explains 19.3% of the variance, revealed a similar motion but of lesser magnitude (Fig. S10).

Despite negatively correlated motions between the two HTH domains, they were never observed in close proximity. The inter-domain space was consistently occupied by a stable accessory module composed of four interacting helical bundles—two each from the IDL (α5, α3) and the C-terminal extension (α11, α12). In this accessory module, as shown in Fig. 2C, the C-terminal extension is positioned between the inter-domain α-helices and the C-HTH domain. The CCA matrix revealed strongly positively correlated motions within this accessory module, akin to those observed within individual HTH domains (Fig. 2E; Fig. S9). Notably, while the accessory module exhibits limited correlated motions with the C-HTH domain, it remains largely uncorrelated with the N-HTH. Overall, our analyses reveal distinctive conformational motions in *w*Zbi-Wmk, where the N-HTH domain moves opposite to both the C-HTH domain and the accessory module, while the latter two show coordinated motions.

### Common features of *w*Zbi-Wmk and *w*Bif-Wmk

**Fig 3:**
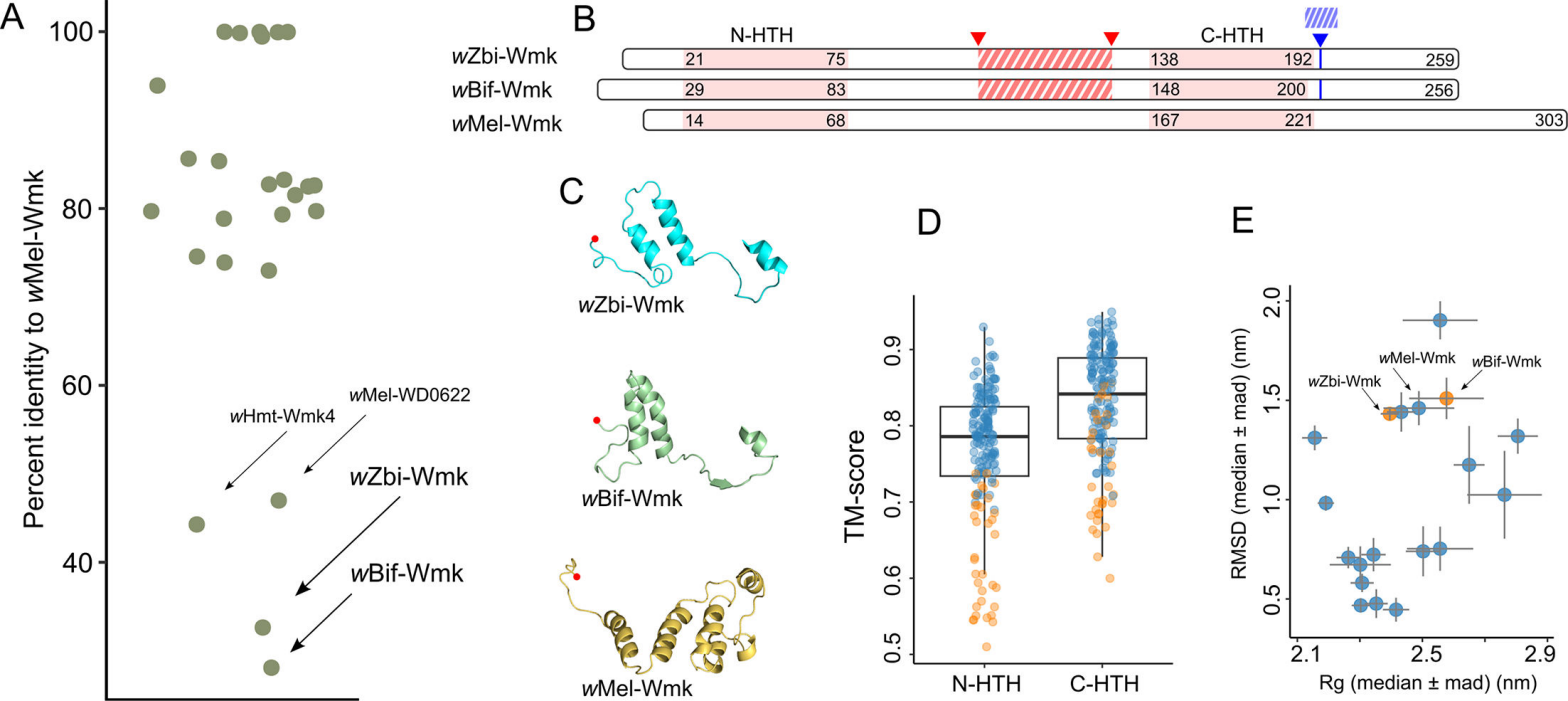
(**A**) Sequence identity between *w*Mel-Wmk and Wmk homologs from 22 *Wolbachia* strains (see Table S4 for the list). (**B**) Simplified graphical presentation of the alignment between *w*Zbi-Wmk, *w*Bif-Wmk, and *w*Mel-Wmk. HTH domains are highlighted in pink. Red and blue patterns indicate sequence deletions and insertions, respectively, relative to *w*Mel-Wmk. Numbers are indicative of residue positions. (**C**) Folds of the IDL of three homologs derived from their representative structures (sampled at 85 ns of the molecular simulation). Red dot suggests N-terminal. (**D**) Box plot showing pairwise alignment of N-HTH and C-HTH domains across all Wmk homologs. Orange color indicates the comparisons involving the distant-Wmks: *w*Zbi-Wmk and *w*Bif-Wmk. Comparisons among type-I-Wmks are in light blue. (**E**) Scatter plot showing the extent of conformational change—measured as RMSD and radius of gyration (Rg)—during MD simulation among Wmk homologs. Each point represents a homolog and shows the median values of the distributions, with the median absolute deviations (mad) indicated by vertical and horizontal lines. All values were calculated from conformations after the initial 20 ns equilibration offset. Orange circles are for the distant-Wmks, and light blue for type-I-Wmks.

We analyzed protein sequences of known Wmk homologs (full length containing two HTH domains) from 22 *Wolbachia* strains to assess sequence divergence of *w*Zbi-Wmk (Table S4). Our comparisons revealed that *w*Zbi-Wmk shares only 33% sequence identity with *w*Mel-Wmk (Fig. 3A; Table S4). This represents a highly divergent Wmk homolog relative to *w*Mel-Wmk. The other well-known example is from the MK *Wolbachia* strain *w*Bif (host: *Drosophila bifasciata*), where *w*Bif-Wmk shows only 26% identity (or 28% based on our analysis) to *w*Mel-Wmk (17). Interestingly, *w*Zbi-Wmk and *w*Bif-Wmk share a higher similarity (*~*48%) than each of them to *w*Mel-Wmk, suggesting either a shared evolutionary origin or convergence at the sequence level.

Multiple sequence alignment revealed that both *w*Zbi-Wmk and *w*Bif-Wmk harbor deletions of approximately 36 and 32 residues, respectively, within the IDL adjacent to the C-HTH domain, as well as insertions of ~7–9 residues immediately downstream of the C-HTH (Fig. 3B; Fig. S11). In addition to these large-scale insertions and deletions, residue-level comparisons of the conservation patterns showed widespread amino acid substitutions across the entire sequence, further distinguishing these two distant homologs from *w*Mel-Wmk and its close homologs (Fig. S12). As per the *wmk* typing introduced by Lefoulon et al. (41), *w*Mel-Wmk and its close homologs (73%–100% identity to *w*Mel-Wmk) in our data set correspond to the Type-I group (Fig. S19). Hereafter, we use the term “type-I-Wmks” to denote this group of homologs. In contrast, highly divergent *wmk* sequences were excluded from the classification in Lefoulon et al. (41) and were not assigned any typing. Here, we use the term “distant-Wmks” to refer to *w*Zbi-Wmk and *w*Bif-Wmk. These distant Wmks might represent additional *wmk* types in *Wolbachia*. Nevertheless, a more comprehensive data set of *wmk* homologs would provide further insights into the classification.

AF2 structural predictions for 19 Wmk homologs from 17 *Wolbachia* strains, evaluated with MD simulations (Fig. S13 and S14), demonstrated how sequence-level divergence translates into structural variation. In agreement with the observed deletions, the IDL of *w*Zbi-Wmk and *w*Bif-Wmk featured only two helical bundles, compared to the four helices found in type-I-Wmks (Fig. 3C; Fig. S14). Likewise, the insertions downstream of the C-HTH in the distant-Wmks resulted in an extended loop (Fig. S15). Aside from these differences, no major structural deviations were observed. Importantly, the functionally critical HTH domains retained a conserved tetra-helical fold across all homologs (median TM-score: 0.78 for N-HTH and 0.84 for C-HTH) (Fig. 3D; Fig. S15), accompanied by a transient three-residue 310-helix. Even the extent of within-protein conformational changes during MD simulations—measured by the RMSD and the radius of gyration (Rg)—was comparable across all homologs (Fig. 3E). For example, the median Rg of type-I-Wmks ranged from 2.16 to 2.8 nm, encompassing the values observed for the distant-Wmks (*w*Bif-Wmk: 2.58; *w*Zbi-Wmk: 2.4). However, due to sequence variation among homologs and the presence of flexible loop regions, each homolog likely explored distinct areas of conformational space during the MD simulations. Consequently, the distribution of Rg values among MD-derived conformations differed markedly among homologs (Pairwise Wilcoxon test: *P* <0.05), except for a few pairs (see Table S5).

### Structural divergence among Wmk homologs

Due to the modular architecture of the Wmk protein and the intrinsic flexibility between its domains, aligning homologous proteins posed significant challenges. Module-wise alignments were insufficient to capture overall structural differences, as individual modules, such as the IDL and the C-terminal extension, appeared to interact closely in their three-dimensional conformations (Fig. 2C; Fig. S15). To account for this molecular flexibility, we employed two complementary approaches for analyzing structural divergence among Wmk homologs. In the first approach, we compared MD-derived representative structures using FATCAT, a tool that accommodates structural flexibility by allowing “twists” in the alignment (42). In the second approach, we sampled eight conformational states per homolog from MD simulations and performed exhaustive pairwise comparisons using TM-align (43).

**Fig 4:**
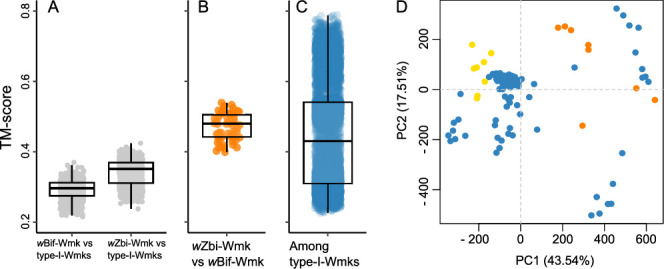
TM-align-based structural alignment (**A**) between type-I-Wmks and the distant-Wmks, (**B**) among distant-Wmks, and (**C**) among type-I-Wmks. Analyses included eight sampled conformations of each homolog from MD simulations. (**D**) Results of PCA of eight MD-sampled conformations from each of 19 Wmk homologs. The plot shows the relationships among conformation along PC1-PC2 axes (type-I-Wmks: blue, *w*Bif-Wmk: orange, *w*Zbi-Wmk: yellow). See Fig. S17 for additional results of PCA.

FATCAT analysis revealed notable structural divergence between type-I-Wmks and the distant-Wmks (Table 1). For instance, *w*Zbi-Wmk and *w*Bif-Wmk showed only 46.67% (range: 43.10–51.06; *P* = 1.09e-06) and 43.88% (range: 33.94–47.19; *P* = 2.04e-05) structural similarity to type-I-Wmks, respectively. These alignments typically required a median of three or four “twists” (range: 3–5) and 18%–22% gaps (range: 15%–28%) to achieve optimal structural matching. Consistent with these results, TM-align analysis of MD-derived conformations showed low median TM-scores of 0.35 (range: 0.24–0.42) for *w*Zbi-Wmk vs. type-I-Wmks and 0.30 (range: 0.22–0.37) for *w*Bif-Wmk vs. type-I-Wmks, indicating substantial conformational differences between the distant-Wmks and type-I-Wmks (Fig. 4A).

**TABLE 1:** FATCAT analysis among representative conformations of Wmk homologs^*a*^

		Similarity (%)	*P*-value	Twists (count)	Gaps (%)	opt-rmsd (Å)
*w*Zbi-Wmk	Type-I-Wmks	46.67 (43.10–51.06)	1.09e-06 (1.65e-07–5.89e-06)	4 (3–5)	18.37 (15.92–28.14)	2.99 (2.43–3.26)
*w*Bif-Wmk	Type-I-Wmks	43.88 (33.94–47.19)	2.04e-05 (1.8e-06–5.47e-04)	3 (3–5)	21.68 (20.22–28.10)	3.05 (2.7–3.81)
*w*Zbi-Wmk	*w*Bif-Wmk	67.21	1.05E-07	2	5.67	3.26
type-I-Wmks	Type-I-Wmks	78.42 (47.17–100)	1.07e-07 (0-1e-05)	4 (1–5)	9.22 (0–27.94)	2.99 (2.07–5.61)

^*a*^ The values are median, and those within parentheses indicate the range (minimum to maximum).

In contrast, members within each group—distant-Wmks and type-I-Wmks—displayed greater structural similarity among themselves, albeit through different patterns. The distant-Wmks (*w*Zbi-Wmk and *w*Bif-Wmk) showed a FATCAT similarity of 67.2% (*P* = 1.05e-07) with only two “twists” and 6% gaps, and a median TM-score of 0.48 (range: 0.40–0.54) (Table 1; Fig. 4B). These matrices suggest an overall structural resemblance between the homologs, with minor variations likely attributable to recent evolutionary divergence. In comparison, the members of the type-I-Wmks group showed a higher median FATCAT similarity of 78.42% (range: 47.17–100; *P* = 1.07e-07), though alignments typically required four “twists” (range: 1–5) and 9% gaps (range: 0%–27.9%). Despite the higher FATCAT similarity, TM-align analysis of MD-sampled conformations yielded a wide-range distribution of TM-score from 0.23 to 0.79 with the median value at 0.43 (Fig. 4C). This broader TM-score distribution, especially the occurrence of lower scores, likely reflects substantial conformational plasticity within individual homologs, diverse conformational states among different homologs of the type-I-Wmks, or both.

To assess conformational variability within each homolog, we plotted the homolog-wise distribution of pairwise TM-scores across MD-derived conformations, for all homologs in type-I-Wmks. The analysis revealed a median TM-score of 0.83 (range: 0.49–0.95), indicating relatively minor structural deviations within individual homologs (Fig. S16). The low density of lower TM-scores further supports the view that within-homolog variability alone does not explain the lower TM-scores observed in between-homolog comparisons. To better capture both within- and between-homolog differences, we next performed PCA on all MD-sampled conformations. The first three principal components explained ~76% of total variance, with PC1 and PC2 accounting for 43.5% and 17.5%, respectively (Fig. S17). Visualization of conformational landscape in PC1-PC2 space revealed that among type-I-Wmks, 12 out of 17 homologs formed the core cluster, indicating structural similarities and limited conformational divergence within this subset (Fig. 4D). Three homologs (*w*Mel-Wmk, *w*Hmt-Wmk3, and *w*Bol1b-Wmk) exhibited limited conformational plasticity and were positioned adjacent to the core cluster (Fig. 4D; Fig. S17). In contrast, two additional homologs*—w*Mel-WD0255 and *w*DacB-Wmk—displayed greater within-homolog variability and were located further from the core, implying distinct conformational behavior.

Progressive removal of these five outlier homologs led to a marked improvement in overall structural similarity among type-I-Wmks (Fig. S16): the median TM-score among remaining homologs increased from 0.43 to 0.54, and the lower bound TM-score improved from 0.23 to 0.44. Together, these results highlight that the modular and flexible architecture of Wmk allows for large-scale conformational changes between type-I-Wmks and the distant-Wmks. Structural differences were also observed within each group, suggesting that Wmk divergence underlies complex and dynamic evolutionary trajectories.

## DISCUSSION

By employing an integrative approach combining genome analysis, structure predictions, and MD simulations, our investigation provides new insights into the evolutionary divergence of the Wmk protein. Although Wmk homologs, particularly 2-HTH containing types, have been identified in *Wolbachia* strains infecting insects across several orders, including Diptera, Lepidoptera, Hymenoptera, and Hemiptera, these homologs typically exhibit high sequence similarity to *w*Mel-Wmk (72%–100% identity). Prior to this study, *w*Bif-Wmk, a homolog from a strain infecting a *Drosophila* species, represented the only known divergent copy of Wmk. Recently, another homolog similar to *w*Bif-Wmk was identified in the *w*Pse N101 strain infecting a different *Drosophila* species (41), about which we have discussed below. Here, we report the identification of a divergent homolog from a novel *Wolbachia* strain associated with a beetle species. This newly identified copy, together with comparative structural analysis of Wmk homologs, allowed us to identify the unique molecular architecture of Wmk and to delineate the common features of divergent homologs that set them apart from the type-I-Wmks.

### Insights into Wmk protein function

Predicted protein models of *w*Zbi-Wmk and 18 other Wmk homologs support a modular architecture of Wmk comprising N-terminal extension, N-HTH domain, IDL, C-HTH domain, and C-terminal extension. Structural similarities of both HTH domains to known transcriptional regulators and the identification of DNA-binding regions within each domain suggest that Wmk functions as a regulatory protein and likely acts as a monomer. The inference of monomeric nature of Wmk is based on the structural characterization of other two-HTH-domain regulatory proteins, including the mycobacteriophage repressor TipsytheTRex (44), human centromere protein CENP-B (45), the transcription factor Pax6 (46), and yeast telomeric protein RAP1 (47). Structural studies of these proteins reveal a common DNA-binding mode: the proteins bind to DNA as a monomer wherein two HTH domains insert into adjacent major grooves, while the IDL interacts with the minor groove, establishing backbone contacts and, in some cases, base-specific bonds (44–47). Disruption of the IDL is therefore expected to affect both the specificity and stability of DNA binding, as experimentally demonstrated for the TipsytheTRex repressor (44).

Given the functional relevance of the IDL, our results highlight Wmk structural and functional distinctiveness. Unlike the shorter IDLs (10–20 residues) in the example proteins above, the IDL in Wmk is markedly longer, comprising ~62–98 residues (Fig. 3B). This extended length corresponds to multiple helices, loops, and turns in Wmk (Fig. 3C), in contrast to the simple loop or short helix configurations in the example proteins. Notably, the IDL in Wmk appears to form close interactions with the C-terminal extension, both in predicted structures and MD-derived conformations (see Results). Whether this IDL-C-terminal module contributes to overall protein stabilization, enhances DNA binding, or serves as a site for protein dimerization remains to be determined. Evaluating the Wmk-DNA complexes with and without the intact IDL may reveal the role of IDL on DNA-binding specificity and stability of Wmk.

Our functional inference is primarily based on the full-length Wmk protein that comprises two HTH domains. However, recent work by Arai et al. (30) reported a single-HTH partial *wmk* variant in *w*Hm-t that exhibited host-killing activity. Subsequently, Lefoulon et al. (41) identified single-HTH variants in additional strains. In light of our proposed model, these findings suggest functional diversification of *wmk*, wherein a partial Wmk variant may achieve efficient DNA binding by forming homodimers, heterodimers, or higher-order multimers, analogous to the single-HTH cI repressor of lambda phage (48). In such a scenario, it will be valuable to check whether the IDL-C-terminal module identified here in the full-length Wmk variant is a conserved structural feature in the dimerized- or polymerized-partial-Wmk variants.

### Evolutionary insights into Wmk divergence

Comparisons among 19 Wmk homologs reveal that the two divergent copies, *w*Zbi-Wmk and *w*Bif-Wmk, show clear structural remodeling characterized by a contraction of the IDL. Specifically, these distant-Wmks lack ~32–36 residues in the IDL relative to type-I-Wmks, resulting in only two helices in the former compared to four in the latter. This resulted in measurable divergence in overall protein folds between these two classes, despite largely conserved domain-level conformations. While the functional implications of these structural distinctions remain to be elucidated, our findings provide a new perspective on Wmk divergence.

Investigations into the genotype-phenotype associations of *wmk* have revealed a complex relationship, leading to multiple hypotheses regarding sequence divergence among type-I-Wmks. One possibility is that the modest sequence variation at the 5′ end of the *wmk* gene (~30–60 nucleotides) reflects adaptation for efficient utilization of host cellular and molecular machinery (31). Alternatively, sequence variation may be shaped by differences in host sex-determination pathways or by co-evolutionary dynamics with host-encoded counteracting factors (17), although direct evidence for these mechanisms is currently lacking. More recently, the antagonistic interaction observed between two tandemly placed *wmk* homologs in *Wolbachia* strain *w*Hm-t suggests that the divergence between these homologs, or likely paralogs, may be driven by a toxin-antitoxin relationship (30).

By contrast, the drivers of extensive divergence between distant-Wmks and type-I-Wmks remain challenging to explain. The hypotheses implicating host molecular machinery or sex-determination systems are less likely explanations, since type-I-Wmks sequences are present in both beetles and flies—the same host groups in which distant-Wmks are currently reported. Yet, species-specific effects cannot be completely excluded. Whether co-evolutionary interactions with co-occurring Wmk homologs or with host-encoded counteracting factors contribute to Wmk divergence remains to be tested. Instead, our analyses of key molecular distinctions between the two Wmk classes support divergent evolution as a plausible mechanism underlying the emergence of distant-Wmks. Phylogenies reconstructed from both protein sequence and structural comparisons (Fig. S18) further consolidate this possibility by revealing monophyly of distant-Wmks with high bootstrap values (>88). We propose that a shift in selective pressures, such as those associated with the acquisition of novel target sites, may have driven the emergence of an ancestral *wmk* variant distinct from the type-I-Wmks. Subsequently, the ancestral variant gave rise to homologs such as *w*Bif-*wmk* and *w*Zbi-*wmk* under host-specific evolutionary constraints.

While our investigation primarily employs computational analyses, the identification of conserved and divergent regions between *wmk* classes provides testable hypotheses for future studies. Structural motifs identified here may underlie variation in host-specific interactions and MK phenotypes.

### Divergent homologs in other *Wolbachia* strains

Recently, three additional divergent Wmk homologs were identified in *w*Inn, *w*Bor, and *w*Pse N101 strains, all of which are known to induce MK in their *Drosophila* hosts (41). Along with one copy of a distant Wmk (intact and containing two HTH domains), these strains also harbor multiple copies of homologs belonging to Type I and Type III clusters (classification as per reference [41]). The divergent Wmk in *w*Pse N101 (MCX3064510) shares 79.51% amino acid identity with *w*Bif-Wmk, but only 48.98% with *w*Zbi-Wmk, and it retains the unique sequence characteristics of distant-Wmks when compared with type-I-Wmks (Fig. S20). In comparison, the divergent homologs in *w*Inn (UID81731) and *w*Bor (MBA8752935)—which are identical to each other—are more similar to *w*Mel-Wmk (52.08% identity) than to either *w*Bif-Wmk (27.81%) or *w*Zbi-Wmk (30.16%). Absence of a DNA-binding site in the C-HTH domain of the *w*Inn and *w*Bor homologs (41) further highlights the complexity of *wmk* gene evolution. Future analyses will be essential to delineate the molecular distinctions of these two homologs relative to the type-I-Wmks, the distant-Wmks, and other known *wmk* types.

### *w*Zbi-beetle interaction

The MK phenotype of *w*Zbi in the host beetle *Z. bicolorata* remains unknown. However, field-based studies have reported a female-biased sex ratio in the beetle populations (49, 50). Although genetic and environmental factors, as well as life-history traits, are major determinants of a species’ primary sex ratio (51, 52), the detection of the *wmk* gene in *w*Zbi suggests a potential role of *Wolbachia* in inducing sex ratio distortion in this species. It would be valuable to investigate the MK phenotype of *w*Zbi, determine its infection frequency across beetle populations, and assess whether host genotype influences *Wolbachia* prevalence. Given that this beetle species is widely used as a biocontrol agent against *Parthenium* weed, it is essential to understand how *Wolbachia* influences beetle population demography through potential sex ratio distortion and to evaluate the long-term ecological consequences of this interaction.

From a mechanistic point of view, beetle hosts provide a unique opportunity to explore the molecular divergence of *Wolbachia* MK. Although the underlying mechanism of *wmk*-associated MK remains unknown, evidence from *w*Bif infection in *D. bifasciata* (20) and *w*Mel-*wmk* transgenic expression in *D. melanogaster* (17) indicates that the MK effect involves DNA damage associated with the DC mechanism in male hosts. However, unlike *Drosophila*, where DC involves hypertranscription of the X chromosome in males, DC in beetles likely involves X chromosome downregulation in females (53) or upregulation in both sexes (54). Moreover, the sex-determination system in many beetle species, including *Z. bicolorata*, is of the XYp type (55), in which the meiotic X and Y chromosomes are held together by a protein scaffold without synapsis pairing (56). The unique sex-determination system and potentially divergent DC mechanism in beetles, compared to those in *Drosophila*, raise the possibility of a mechanistic divergence of *wmk*-associated MK.

## MATERIALS AND METHODS

### Genome assembly

In our previous investigation, during the de novo genome assembly of the host beetle *Z. bicolorata* using Nanopore long reads (32), we screened the assembled contigs against the standard database in Kraken v2.1.2 (57). We identified 11 of these contigs as *Wolbachia* origin. The contigs were further confirmed for their origin by similarity check against the NCBI non-redundant prokaryotic database. Subsequently, we used these 11 contigs to filter *Wolbachia*-specific reads from the original error-corrected Nanopore long-reads and Illumina short-reads using Minimap2 v2.24 (58) and bwa-mem2 v2.2.1 (59), respectively. The filtered long reads were assembled using Flye v2.8.1 (--meta) (60) and B-assembler (estimated assembly size = 1.4 Mb, based on the Flye result) (61). The final assembly was error-corrected with the short reads using Polca from MaSuRCA v4.1 (62) in default mode. We confirmed the assembly circularity by checking the assembly graphs from Flye and evaluated the assembly completeness using BUSCO v5.5 (Rickettsiales_odb10) (63) and contamination check with CheckM v1.2.2 (64).

### Search for a plasmid

We verified the resulting assembly graph files, from Flye and B-assembler, for the presence of plasmid-like elements using the bandage v0.9.0 (65). To account for the loss of reads due to the inclusion of *Wolbachia*-specific reads only in the assembly, we retrieved long reads and short reads that did not map to the host beetle genome. Using this larger non-specific data set, we re-assembled the genome and other extra-chromosomal elements with Flye v2.8.1 (using long reads only) (60) and Plassembler v1.6.2 (using both long and short reads) (66) in default modes. We expected that the analyses would result in multiple circular assemblies of varying sizes providing signatures for the presence of putative plasmids. In addition, Plassembler compares the assembled contigs against a curated plasmid database, PLSDB (67). Based on these results, which indicated the absence of independent plasmid-like assembly, we further checked for the presence of plasmid-associated proteins in the *Wolbachia* genome assembly. For this, we retrieved the sequences of known *Wolbachia* plasmids of *w*AlbA (pWALBA1 and pWALBA2) (68), used them to search against our genome using blastn, and filtered the results to retrieve homologies with 80% similarity and 50% query coverage.

### Supergroup classification

We followed the phylogeny-based approach for supergroup identification of the assembled *Wolbachia* genome. First, we compiled a comprehensive data set of 241 *Wolbachia* genomes by combining a previously curated 199 genomes (69), and 42 additional high-quality genomes from NCBI (checkM: minimum 91% completeness and maximum 4% contamination). Our genome, along with 241 *Wolbachia* genomes representing eight supergroups, was used to identify the BUSCO gene markers against the Rickettsiales_odb10 data set using BUSCO v5.5 (63). By using 350 BUSCO gene markers that span at least 95% of the taxa in the data set, we prepared the alignment file using the BUSCO phylogenomics pipeline v20240919 (available at https://github.com/jamiemcg/BUSCO_phylogenomics). This pipeline used MUSCLE v5.3 (70) and trimAI v1.5 (71) to prepare a concatenated supermatrix of 121,171 amino acids long alignment. Use of BUSCO markers ensured that the genes used for phylogeny are of core genetic elements and are most likely devoid of the prophage and repeat regions, which often have distinct evolutionary histories compared to the core of the genome. We reconstructed a phylogenetic tree with IQ-TREE v2.1.4 (72) using the LG + G4 model. 1,000 bootstrap iterations were conducted to assign nodal supports. Based on the resulting tree topology, we assigned supergroup classification to the novel strain. Additionally, we measured GC content of the 127 circular genomes, out of 242, using Seqkit v2.4.0, to verify the genome clustering on the basis of GC percentage (following Vancaester and Blaxter [69]).

### Comparison of genomes

To further characterize the assembled genome, we compared it with two phylogenetically closely related strains: *w*Tae (host: *Sphaerophoria taeniata*) and *w*Con (host: Rhinocyllus conicus). First, we performed orthologous analysis using OrthoVenn3 (73). We then conducted pairwise genome comparisons by calculating average nucleotide identity (ANI) with fastANI v1.33 (74), and used the results to build a synteny map with pyGenomeViz v0.4.4. To estimate genomic divergence and differences in GC content, we used *in silico* DNA-DNA hybridization via GGDC v3.0 (75). Additionally, we employed D-Genies (76) to generate dot plots of pair-wise genome alignments, providing a visual representation of sequence similarity and structural variation among the genomes.

### Genome annotation

We performed genome structural and functional annotation using NCBI Prokaryotic Genome Annotation Pipeline (PGAP), which combines ab initio gene prediction with homology-based methods (77). The resulting coding sequences (CDS) were used to evaluate assembly completeness with BUSCO v5.5 (63). We identified the region of putative replication origin (oriC) by using the Ori-Finder web server (78) with default options (78). To identify genome-wide insertion sequences (IS), we performed a profile HMM-based IS detection with digIS v1.2 (79). To delineate the genes associated with the host reproductive manipulations, we performed a blast-based or MMseqs2-based gene search using previously identified genetic regions across multiple *Wolbachia* genomes as query sequences. Cif from *w*Mel (CifA: WP_010962721, CifB: WP_010962722), Piff from *w*For (WZK11886), Oscar from *w*Fur (BDC30318), and Wmk from multiple sources (*w*Mel: AAS14223 and AAS14326; *w*Inn: QEQ51096; *w*Bor: QEQ51099; wBif: QEQ51101; Multispecies: WP_010962718, WP_038198911, and WP_010962645) were used for the references.

### Phage characterization

We used the Phaster web server (80) to identify the putative regions of phage WO in the *Wolbachia* genome assembly. The Phaster annotation was augmented by further localizing the WO elements through homology search (tblastn) against representative phage WO from four different srWO clades as classified in Bordenstein and Bordenstein (35). We considered WOCauB3 (AB478516.1: B3gp1-GF2gp25), WOVitA1 (HQ906662.1), WOMelB (AE017196.1: WD_0563-WD_0648), WOFol3s2 (CP015510.2: ASM33_05515-ASM33_05155), and the Octomom (AE017196.1: WD_0507-WD_0514) for the homology search. As a result, we ensured inclusion of all defined phage regions like core modules, EAM, WO-PC1, WO-PC2, the Undecim cluster, and the Octomom in the database. The Phaster annotation, along with the homology search, served as the seed locations to manually inspect the presence of phage WO genes through the “walking out” method (following [35]). Annotations to the phage regions were further complemented by PGAP analysis and manual search against the NR-cluster. The identified phage WO was further classified based on phylogenetic clustering using the protein sequence of Serine recombinase from our assembly and that from the described WO classes. Here, we used the aligned sequences and the best-fit model for the maximum likelihood tree reconstruction with 1,000 bootstraps for nodal support in iqtree v2.3.6 (72).

### Comparative gene analysis

We compared Wmk from *w*Zbi with 26 additional homologs from 22 *Wolbachia* strains. These additional homologs contained two HTH domains each and were identified previously in Perlmutter et al. (17) and Arai et al. (30). To assess sequence similarity, we computed amino acid identities of all homologs relative to the reference Wmk from *w*Mel using MMseqs2 v16.747c6 (81). Based on sequence homology, we designated the Wmk proteins from *w*Zbi and *w*Bif as “distant” homologs of *w*Mel-Wmk. The remaining sequences, except for two, were classified as “close” homologs, with sequence identities to *w*Mel-Wmk ranging between 72% and 100%. Two homologs (*w*Hmt-Wmk4: BDG76735 and *w*Mel-WD0622: AAS14323), which were divergent to both *w*Mel-Wmk (<47%) and the distant homologs (<35%), were excluded from further analysis. Additionally, five close homologs were removed due to redundancy, as they were duplicates of either *w*Mel-Wmk or *w*Bor-Wmk.

The final data set comprised 20 Wmk homologs from 18 *Wolbachia* strains. These were aligned using Clustal Omega (via the UniProt “Align” tool) and M-Coffee (T-Coffee v11 online server) (82) to assess how the two distant homologs differed from the remaining sequences. To further validate the unique sequence composition of the distant homologs, we computed residue-wise conservation scores. For this, we generated two datasets—one including all 20 sequences and another excluding the distant homologs—and aligned them separately using M-Coffee. Residue-wise conservation scores were then calculated with the Shannon-entropy estimation method (83). Scores from both alignments were mapped onto a common reference sequence, *w*Mel-Wmk, and only residue positions 10 to 268—selected to exclude alignment-end ambiguities—were considered for comparison. The differences in conservation scores between the two datasets were plotted to identify regions with altered conservation patterns, thereby highlighting segments uniquely divergent in the distant homologs.

### Protein folding and MD

We used ColabFold v1.5.5 (84) to predict protein structure from the sequence. ColabFold is based on MMseqs2 (81) for multiple sequence alignment and AlphaFold2 (33) for protein structure predictions. The jobs for structure prediction were submitted using Colab notebook with its default settings (num_models: 5, num_recycles: 3). We successfully predicted the structures for 19 Wmk homologs from 17 *Wolbachia* strains. The output files were evaluated with the help of ChimeraX v1.8 (85), PAE viewer (86), and PyMOL v2.6 (OS-build; https://github.com/schrodinger/pymol-open-source). We simulated MD of the predicted protein models in GROMACS v2023.4 by following the standard instructions described in Lemkul (34). Briefly, we retrieved the best-fit model of each protein, embedded it in a solvated, electroneutral system, and minimized the energy state of the structure. We then checked the assembled system for equilibration with respect to temperature and pressure and allowed MD simulation by releasing positional constraints and applying the all-atom OPLS force field. We employed tools like VMD v1.9.4 (87), QtGrace v0.2.6 (https://sourceforge.net/projects/qtgrace/), PyMol v2.6 (OS-build), and TM-align v20220412 (43) to analyze the simulation outputs.

### Principal component analysis

We conducted PCA using the R package Bio3d v2.4-5 (88). First, we performed PCA on MD-derived conformations of *w*Zbi-Wmk. The trajectory file from the MD simulation was converted to “dcd”’ format with VMD v1.9.4, considering the first 20 nanoseconds (ns) as the equilibration offset. All trajectory frames were then superposed on the C-alpha atoms of the representative conformation of *w*Zbi-Wmk (extracted at 85 ns of the simulation), and the resulting aligned matrix was subjected to PCA. Additionally, a cross-correlation analysis was performed on the same matrix to calculate the pairwise cross-correlation coefficients between residues.

In a separate analysis, we performed PCA on MD-sampled conformations of 19 Wmk homologs. For each homolog, eight conformations were sampled between 25 and 95 ns of the simulation at 10 ns intervals. Using multiple sequence alignment, all conformations were aligned together to determine residue-residue correspondences. Then, the structures were iteratively superposed to identify a set of “core” residues exhibiting minimal positional variability. Based on the resulting core residue identification graph, 62 residues (positions 8–51 and 53–70) were selected as the “core” set. These residues span the N-HTH domain, supporting their use as the structural anchor during superposition. The resulting aligned matrix of the superposition, with gaps removed, was used for PCA.

### Wmk phylogenetic analyses

We conducted a phylogenetic analysis of 20 Wmk homologs from 18 *Wolbachia* strains. Amino acid sequences were aligned using mafft v7.490 (89) in “auto” mode. The resulting alignment was used to analyze the best-fit model and maximum likelihood tree using iqtree v2.1.4 (72). Nodal supports were derived from 1,000 bootstrap analyses. For structure-based phylogeny, we analyzed the representative protein models of 19 Wmk homologs from 17 *Wolbachia* strains and followed a recent model-based analysis described in Garg and Hochberg (90). Briefly, we extracted 3Di sequences from the PDB files using Foldseek v9.427df8a (91). The 3Di sequences were aligned using mafft v7.490 with the G-INS-I model and the 3Di scoring matrix from Foldseek. The alignment file was then trimmed by trimAl v1.5 (71), and subsequently tested for the best-fit model among GTR20, Q3DiAF, and Q3DiLLM (90). Using IQ-TREE 2 v2.1.4, we analyzed the maximum likelihood tree by incorporating the best-fit model (Q3DiAF) and 1,000 bootstraps.

## Data Availability

The assembled genome of wZbi has been deposited to NCBI-GenBank and is accessible with the accession number CP149530. It accompanies the annotation provided by the NCBI Prokaryotic Genome Annotation Pipeline (PGAP). The code for the analyses presented in this paper is available at https://github.com/sahoo-rk/wZbi. Additional information and resources may be accessed at https://doi.org/10.6084/m9.figshare.30736040 or obtained from the lead contact (Ranjit Kumar Sahoo: sahoo_rk@outlook.com).

## SUPPLEMENTAL MATERIAL

Supplemental material spectrum.02893-25-s0001.pdf Tables S1 to S5 and Figures S1 to S20.

Video S1 spectrum.02893-25-s0002.mp4 The video shows conformational transition between two MD-derived *w*Zbi-Wmk conformations C1 (start) and C7 (end). The conformations were sampled at 25 ns and 85 ns, respectively, and are anchored at the N-HTH domain (residue 21-75) to highlight the structural flexibility among them, as mentioned in Fig. S8. The intermediate states between the C1 and C7 are predicted using 'morph' function in ChimeraX v1.8.

ASM does not own the copyrights to Supplemental Material that may be linked to, or accessed through, an article. The authors have granted ASM a non-exclusive, world-wide license to publish the Supplemental Material files. Please contact the corresponding author directly for reuse.
