# Supplementary material for: Genome assembly and protein structure modeling reveal key molecular features of divergent wmk homologs in Wolbachia
Source: Microbiol Spectr. 2025 Dec 30;14(2):e02893-25. doi: 10.1128/spectrum.02893-25 (PMC12889037; doi:10.1128/spectrum.02893-25)
Supplement: Supplemental material — Tables S1 to S5 and Figures S1 to S20. [file spectrum.02893-25-s0001.pdf]

Supplementary Information for the title "Genome assembly and protein structure modelling reveal key molecular features of divergent *wmk* homologs in *Wolbachia*".

Table S1: BUSCO (whole genome and annotated proteins) and checkM (whole genome) assessments of *w*Zbi.

**BUSCO**	Rickettsiales_odb10 (n=364)		Alphaproteobacteria_odb10 (n=432)	
	Genome	Proteins	Genome	Proteins
Complete	99.1	99.1	77.1	77.5
Single-copy	98.6	98.6	76.9	77.3
Duplicated	0.5	0.5	0.2	0.2
Fragmented	0.5	0	2.3	1.6
Missing	0.4	0.9	20.6	20.9
**checkM** (o_Rickettsiales (UID3811))	Genome	-	-	-
Completeness	99.79	-	-	-
Contamination	0.75	-	-	-
Strain heterogeneity	40.00	-	-	-

Table S2: Multi-locus Sequence Typing (MLST) and WSP-based sequence typing of *w*Zbi through PubMLST database for *Wolbachia* spp. The closest MLST allelic match (*) reflects changes at 1-4 nucleotides per allele. The MLST profile based on these allelic types finds ST-480 as the nearest match to *w*Zbi (differ in ftsZ typing with ftsZ: 230) in the database (last checked on July 25, 2025).

	Allelic type (*closest match)				
MLST	fbpA: 36*	ftsZ: 75*	coxA: 33*	gatB: 32*	hcpA: 42
WSP	HVR1: 52	HVR2: 57	HVR3: 39	HVR4: 255*	-

Table S3: Results of *wmk* gene annotation through database search using CDD-search (against NCBI-CDD database) and ScanProSite (against ProSite database).

		CDD-search						ScanProSite			
	**domain**	**interval**	**superfamily**	**accession**	**desrciption**	**e-value**		**range**	**note**	**score**	**motif**
*w*Zbi-Wmk	N-domain	16-76	XRE	COG1476	XRE-family	3.88E-14		21-75	cro/C1-type	16.046	32-51
	C-domain	128-200	HipB	COG1396	XRE-family	5.44E-09		138-192	cro/C1-type	10.994	149-168
*w*Bif-Wmk	N-domain	28-81	HTH_XRE	smart00530	XRE-family	3.21E-08		29-83	cro/C2-type	12.595	40-59
	C-domain	149-208	HipB	COG1396	XRE-family	2.20E-05		148-200	cro/C3-type	9.378	159-178
*w*Mel-Wmk	N-domain	5-84	HipB	COG1396	XRE-family	2.29E-14		14-68	cro/C4-type	14.757	25-44
	C-domain	162-235	HipB	COG1396	XRE-family	1.25E-14		167-221	cro/C5-type	14.041	178-197

Table S4: Sequence similarity analysis of Wmk homologs across *Wolbachia* strains. MMseqs2-based sequence similarity was computed between *w*Mel-Wmk (AAS14326) and 26 homologs from 23 *Wolbachia* strains. Each strain is annotated with its phenotypes as either MK (male killing) or UMK (male killing status unknown), based on observations in natural or trasinfected insect hosts. A subset of 20 homologs from 18 strains was selected for multiple sequence alignment, as mentioned in the last column 'msa'.

accession	strain	host species	host order	fident	alnlen	mismatch	evalue	strain phenotype	msa
AAS14326.1	*w*Mel	*Drosophila melanogaster*	Diptera	1	303	0	0	UMK	yes
AGJ99709.1	*w*Ha	*Drosophila simulans*	Diptera	1	303	0	4.3E-187	UMK	no
ACN95365.1	*w*Ri	*Drosophila simulans*	Diptera	1	303	0	4.3E-187	UMK	no
ERN55632.1	*w*MelPop	*Drosophila melanogaster*	Diptera	1	303	0	4.3E-187	pathogenic	no
CAOU02000042.1 (266:1177)	*w*Suzi2	*Drosophila suzukii*	Diptera	1	303	0	4.3E-187	UMK	no
EAL58516.1	*w*Ana	*Drosophila ananassae*	Diptera	1	303	0	4.3E-187	UMK	yes
AOV87449.1	*w*IncCu	*Drosophila incompta*	Diptera	0.996	303	1	2.1E-186	UMK	yes
JQAM01000018.1 (519:1430)	*w*Rec	*Drosophila recens*	Diptera	0.996	303	1	2.8E-186	MK	yes
LYUY01000031.1 (703:1598)	*w*Nfe	*Nomada ferruginata*	Hymenoptera	0.939	297	18	2.5E-172	UMK	yes
CAOH01000056.1 (9781:10692)	*w*Bol1b	*Hypolimnas bolina*	Lepidoptera	0.858	303	43	3.1E-161	MK	yes
CAQ54347.1	*w*Pip	*Culex quinquefasciatus*	Diptera	0.855	305	44	3.2E-158	UMK	yes
LYUX01000014.1 (18117:19034)	*w*Npa	*Nomada panzeri*	Hymenoptera	0.829	305	52	6.0E-158	UMK	yes
QER90991.1	*w*CauB	*Cadra cautella*	Lepidoptera	0.832	305	51	9.3E-156	MK	yes
CDR78644.1	*w*Au	*Drosophila simulans*	Diptera	0.826	306	53	1.9E-153	UMK	yes
AAQP01000017.1 (2974:3894)	*w*Wil	*Drosophila willistoni*	Diptera	0.826	306	53	1.9E-153	UMK	yes
OAM04567.1	*w*DacB	*Dactylopius coccus*	Hemiptera	0.815	303	56	5.7E-151	UMK	yes
CCE77454.1	*w*AlbB	*Aedes albopictus*	Diptera	0.788	303	63	3.4E-149	UMK	yes
OAM03042.1	*w*DacA	*Dactylopius coccus*	Hemiptera	0.794	302	61	1.0E-143	UMK	yes
QEQ51096.1	*w*Inn	*Drosophila innubila*	Diptera	0.797	297	59	6.0E-142	MK	no
QEQ51099.1	*w*Bor	*Drosophila borealis*	Diptera	0.797	297	59	6.0E-142	MK	yes
AAS14324.1	*w*Mel	*Drosophila melanogaster*	Diptera	0.746	296	74	2.7E-137	UMK	yes
BDG76734.1	*w*Hmt	*Homona magnanima*	Lepidoptera	0.729	296	79	3.4E-133	MK	yes
AAS13997.1	*w*Mel	*Drosophila melanogaster*	Diptera	0.74	293	75	6.4E-133	UMK	yes
BDG76735.1	*w*Hmt	*Homona magnanima*	Lepidoptera	0.443	319	164	2.78E-70	MK	no
AAS14323.1	*w*Mel	*Drosophila melanogaster*	Diptera	0.47	295	154	3.04E-68	UMK	no
QEQ51101.1	*w*Bif	*Drosophila bifasciata*	Diptera	0.28	262	168	5.87E-21	MK	yes
WKH12_02010	*w*Zbi	*Zygogramma bicolorata*	Coleoptera	0.326	264	159	1.91E-33	UMK	yes

Table S5: Pairwise Wilcoxon Test (also known as Mann-Whitney test) comparing radius of gyration across 19 Wmk homologs. Conformational states for each Wmk homolog were derived from molecular dynamics simulations after applying a 20-ns equilibration offset. The table reports the P-values from all pairwise Wilcoxon rank-sum comparisons, evaluating differences in median radius of gyration (Rg) between homologs. Lower P-values ($P < 0.05$) indicate significant differences in median Rg. Red-highlighted cells denote comparisons with no significant difference between the pairs..

	AAQP01000017	AAS13997	AAS14324	AAS14326	AOV87449	BDG76734	CAOH01000056	CCE77454	CDR78644	EAL58516	JQAM01000018	LYUX01000014	LYUY01000031	OAM03042	OAM04567	QEQ51099	QER90991	QEQ51101
**AAS13997**	8.89E-263	NA	NA	NA	NA	NA	NA	NA	NA	NA	NA	NA	NA	NA	NA	NA	NA	NA
**AAS14324**	5.94E-215	3.25E-257	NA	NA	NA	NA	NA	NA	NA	NA	NA	NA	NA	NA	NA	NA	NA	NA
**AAS14326**	1.20E-47	4.98E-258	1.75E-49	NA	NA	NA	NA	NA	NA	NA	NA	NA	NA	NA	NA	NA	NA	NA
**AOV87449**	6.50E-127	8.89E-263	7.63E-255	8.40E-139	NA	NA	NA	NA	NA	NA	NA	NA	NA	NA	NA	NA	NA	NA
**BDG76734**	1.03E-262	1.40E-31	2.66E-162	1.62E-215	9.06E-263	NA	NA	NA	NA	NA	NA	NA	NA	NA	NA	NA	NA	NA
**CAOH01000056**	2.62E-09	8.89E-263	3.82E-195	4.70E-32	1.84E-160	9.62E-263	NA	NA	NA	NA	NA	NA	NA	NA	NA	NA	NA	NA
**CCE77454**	8.89E-263	8.89E-263	8.89E-263	9.51E-263	1.12E-262	8.89E-263	8.89E-263	NA	NA	NA	NA	NA	NA	NA	NA	NA	NA	NA
**CDR78644**	3.30E-153	8.89E-263	4.28E-56	3.22E-03	2.95E-240	1.70E-254	9.04E-122	8.89E-263	NA	NA	NA	NA	NA	NA	NA	NA	NA	NA
**EAL58516**	8.89E-263	8.89E-263	8.89E-263	1.66E-262	3.43E-262	8.89E-263	8.99E-263	2.40E-58	8.89E-263	NA	NA	NA	NA	NA	NA	NA	NA	NA
**JQAM01000018**	2.75E-261	8.89E-263	8.89E-263	2.33E-249	1.16E-236	8.89E-263	1.41E-261	7.50E-189	8.93E-263	3.85E-195	NA	NA	NA	NA	NA	NA	NA	NA
**LYUX01000014**	1.31E-238	8.89E-263	8.89E-263	1.70E-206	1.08E-111	8.89E-263	5.80E-249	3.15E-262	2.40E-262	6.60E-259	4.22E-134	NA	NA	NA	NA	NA	NA	NA
**LYUY01000031**	5.65E-130	8.89E-263	2.50E-252	2.43E-165	5.52E-31	9.03E-263	2.98E-154	6.45E-206	2.66E-234	4.88E-214	1.32E-41	5.82E-01	NA	NA	NA	NA	NA	NA
**OAM03042**	8.91E-181	8.89E-263	1.14E-262	5.04E-167	1.80E-08	8.89E-263	4.52E-212	6.84E-262	6.29E-259	1.10E-259	4.06E-195	1.02E-64	6.26E-15	NA	NA	NA	NA	NA
**OAM04567**	1.47E-178	4.00E-157	5.81E-01	1.33E-41	3.59E-244	4.18E-105	1.88E-156	8.89E-263	7.70E-40	8.89E-263	8.93E-263	4.12E-262	1.92E-240	4.04E-258	NA	NA	NA	NA
**QEQ51099**	8.91E-253	2.72E-262	1.04E-31	2.03E-121	2.98E-261	2.29E-111	9.69E-249	8.89E-263	4.51E-171	8.89E-263	8.89E-263	8.89E-263	1.37E-260	9.16E-263	2.33E-28	NA	NA	NA
**QER90991**	5.11E-240	8.89E-263	8.89E-263	5.90E-208	9.95E-118	8.89E-263	2.22E-249	5.55E-262	2.37E-262	4.25E-259	6.41E-142	6.03E-01	5.99E-01	2.37E-67	3.73E-262	8.89E-263	NA	NA
**QEQ51101**	4.58E-234	1.11E-145	7.05E-10	1.58E-74	2.96E-258	4.28E-81	9.04E-222	8.89E-263	1.43E-85	8.89E-263	8.89E-263	8.89E-263	1.35E-256	9.20E-263	1.09E-07	3.65E-05	8.89E-263	NA
**WKH12**	1.49E-29	8.89E-263	5.46E-248	5.10E-85	7.70E-92	1.05E-262	2.19E-64	8.89E-263	5.57E-214	8.89E-263	1.66E-262	4.50E-246	1.85E-93	2.51E-151	2.96E-221	2.79E-259	2.00E-244	5.96E-254

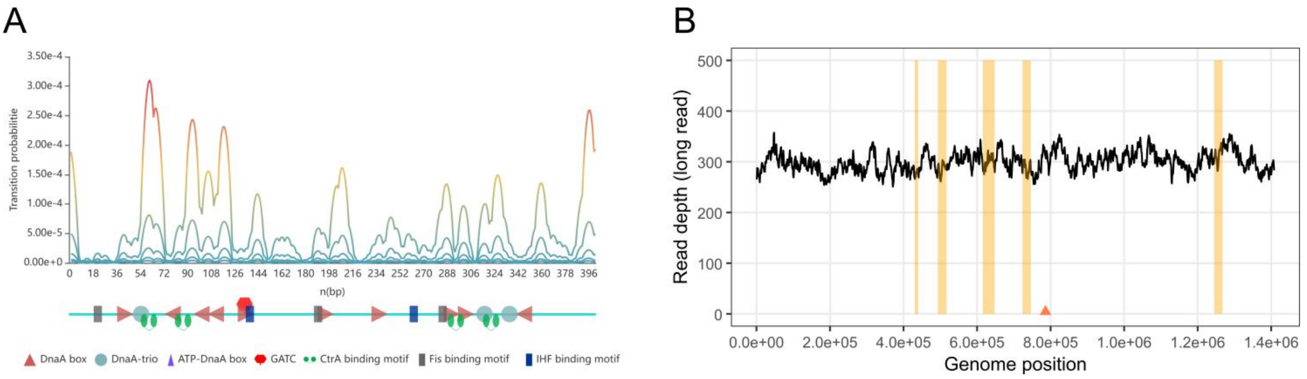

Figure S1: (A) Annotation of the putative origin of replication (oriC) in *w*Zbi using OriFinder2022. The predicted oriC spans 401 bp (position: 785, 898 - 786, 298) and corresponds to the region highlighted in Fig. 1A. (B) Genome-wide read depth profile generated by mapping long-read sequencing data to the *w*Zbi assembly. Orange bars represent regions corresponding to WO phage elements, and the coral-colored triangle marks the location of the predicted OriC.

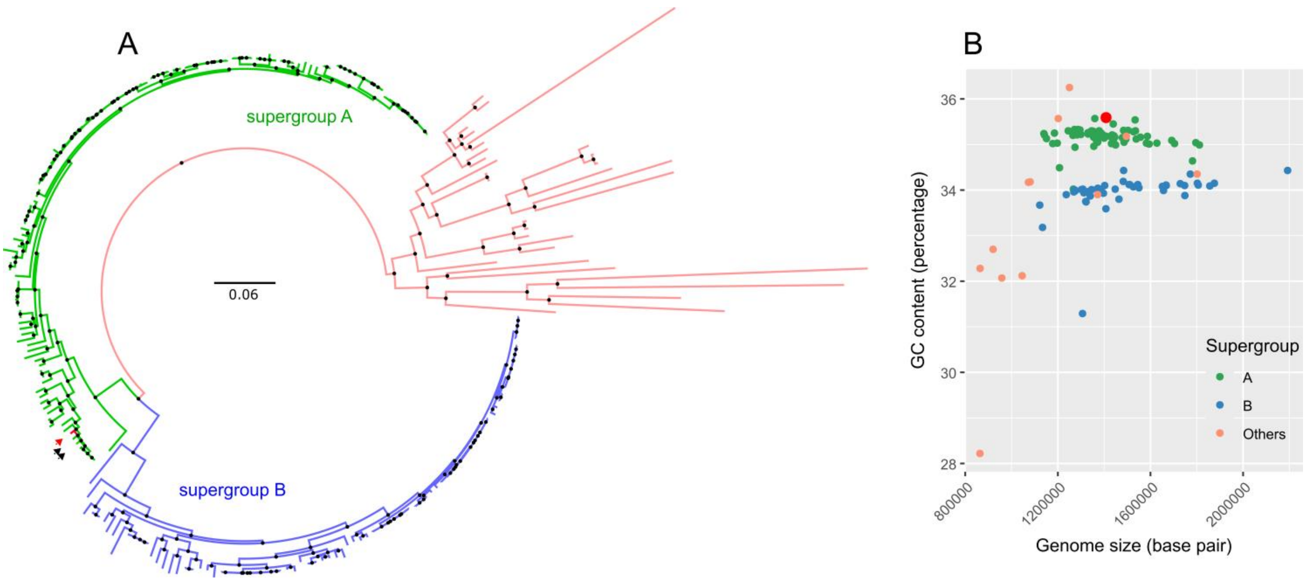

Figure S2: (A) Maximum likelihood based unrooted phylogenetic tree of 242 *Wolbachia* strains, reconstructed from 350 shared BUSCO gene markers. For visualization, the tree was rooted at the midpoint of the clade containing supergroups A and B and the remaining strains. Node support values are indicated by circle sizes, based on 1000 bootstrap replicates. The *w*Zbi is highlighted with a red branch and a red arrow. Black arrows indicate two other strains, *w*Tae and *w*Con (see Fig. S3 for detail). (B) The relationship between genome size and GC content across 126 complete *Wolbachia* genomes. The value for *w*Zbi is highlighted as an enlarged red dot.

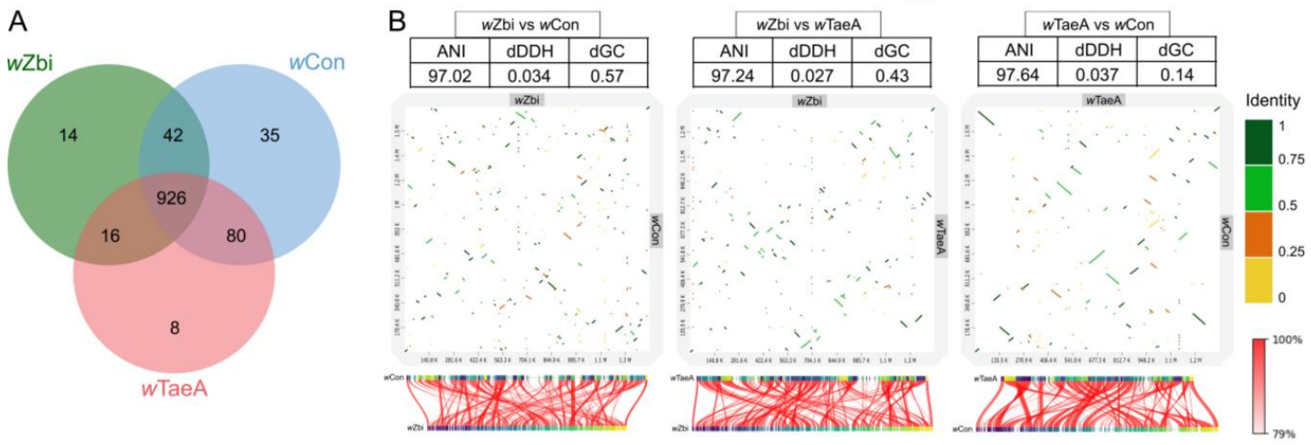

Figure S3: Comparative genomics of *w*Zbi, *w*Tae, *w*Con. (A) Venn diagram showing the number of shared and unique orthogroups among three closely related *Wolbachia* genomes: *w*Zbi (host: *Zygogramma bicolorata*), *w*TaeA (GCA_947250735; host: *Sphaerophoria taeniata*) and *w*Con (GCA_947250775; host: *Rhinocyllus conicus*). The genomes are phylogenetically closely related and are marked with arrows in Fig. S2A. (B) Pairwise genome comparisons based on three matrices: average nucleotide identity (ANI), in silico DNA-DNA hybridization (dDDH), and difference in GC content (dGC). D-Genies dot plots display pairwise genomic alignments, where scattered dots indicate extensive genome rearrangements and lack of synteny. This is further supported by the synteny maps from pyGenomeViz (bottom panel), which used pairwise ANI scores to illustrate synteny breakdown across genomes.

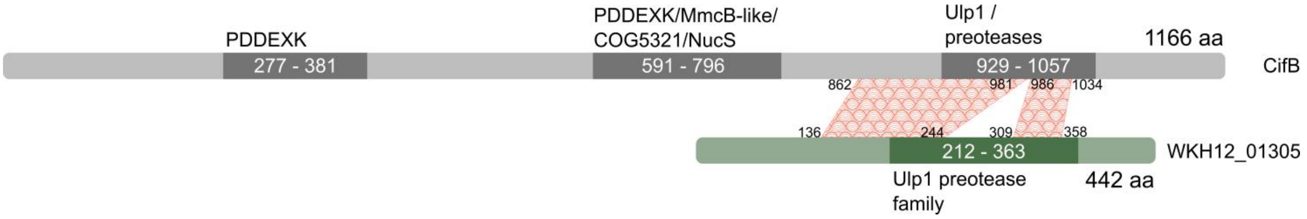

Figure S4: Comparison between putative CifB homolog in *w*Zbi and the reference protein. The top gray bar represents the reference CifB protein sequence (WP_010962722.1), annotated according to Lindsey et al., 2018. The bottom green bar denotes the putative CifB homolog in *w*Zbi (gene ID: WKH12_01305). Protein domains in WKH12_01305 were identified using the NCBI Conserved Domain Database (CDD). Areas connecting the bars indicate regions of similarity identified by BlastP: left-side area shows 31% identity ($E=2e^{-12}$) and the right-side area shows 42% identity ($E=3e^{-08}$). Numbers along the bars represent amino acid positions.

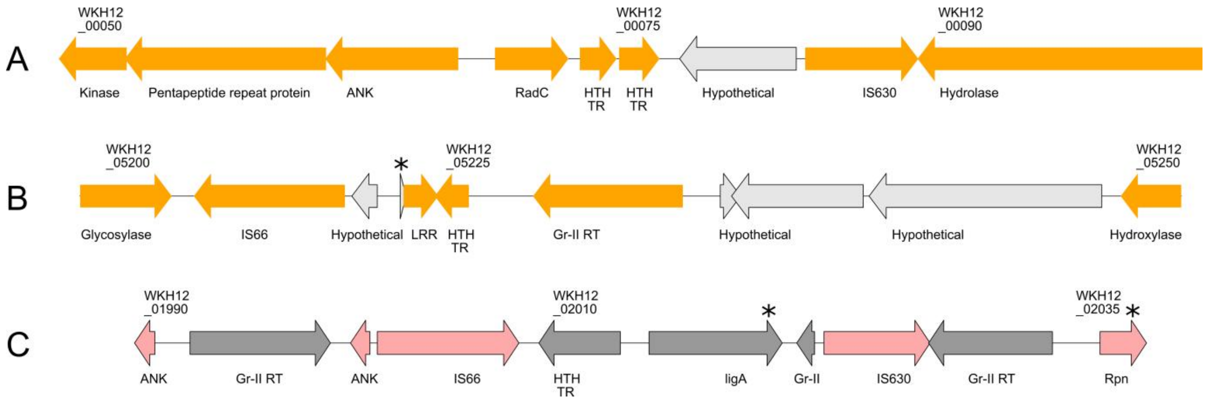

Figure S5: Genome localization maps of three putative Wmk homologs (WKH12_00075, WKH12_05225, and WKH12_02010) identified in *w*Zbi. Panels (A) and (B) show genomic neighborhoods surrounding WKH12_00075 and WKH12_05225 loci, respectively. In these panels, orange colored regions indicate annotated gene names, while light grey regions represent coding sequences with hypothetical functions. Panel (C) shows the organization of the WOZbi1 region and corresponds to Fig. 1B and Fig. 2A. In this panel, pink color denotes transposases (e.g., IS66, IS630) and dark grey represents non-core genes. Star symbols across all panels mark pseudogenes.

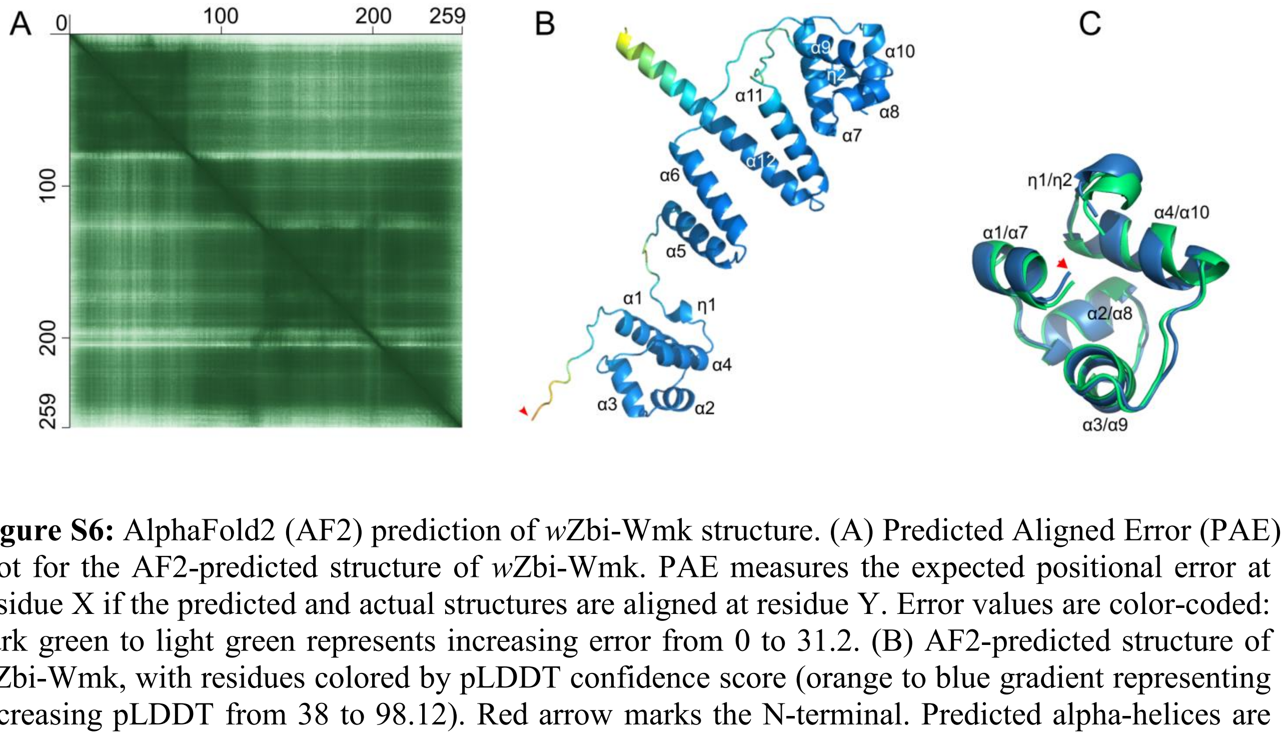

Figure S6: AlphaFold2 (AF2) prediction of *w*Zbi-Wmk structure. (A) Predicted Aligned Error (PAE) plot for the AF2-predicted structure of *w*Zbi-Wmk. PAE measures the expected positional error at residue X if the predicted and actual structures are aligned at residue Y. Error values are color-coded: dark green to light green represents increasing error from 0 to 31.2. (B) AF2-predicted structure of *w*Zbi-Wmk, with residues colored by pLDDT confidence score (orange to blue gradient representing increasing pLDDT from 38 to 98.12). Red arrow marks the N-terminal. Predicted alpha-helices are labeled. (C) Structural alignment between the two HTH domains of AF2-predicted model (rmsd=0.7Å). Sky blue color represents the N-HTH domain and light green color represents the C-HTH domain. Overlapping alpha-helices are labeled, and the red arrow indicates the N-terminal.

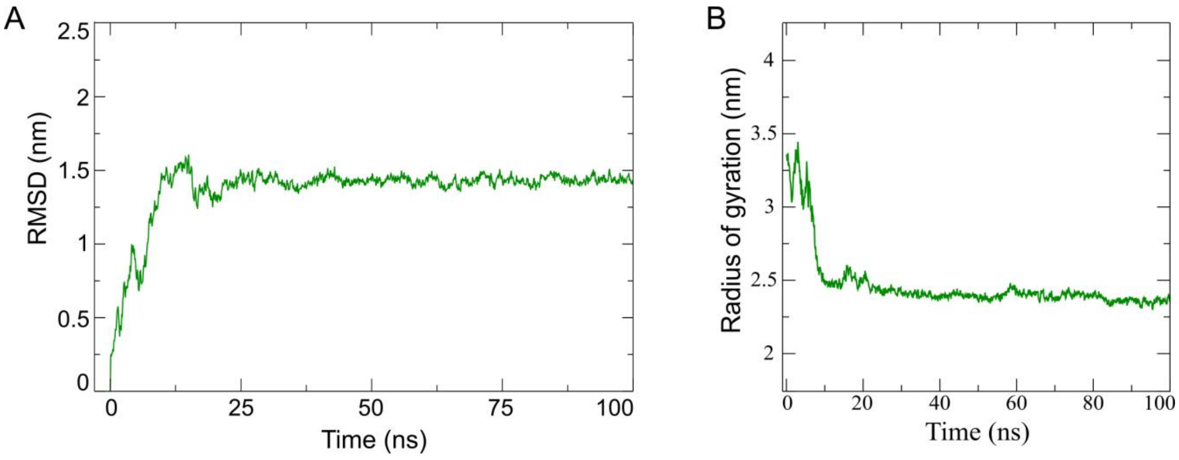

Figure S7: Molecular dynamics (MD) simulation of the AF2-predicted *w*Zbi-Wmk structure. Root-mean-square-deviation (RMSD) plot (in A) and radius of gyration (rg) plot (in B) show changes in overall conformation relative to the initial AF2-predicted structure, and the compactness of simulated protein states over time, respectively.

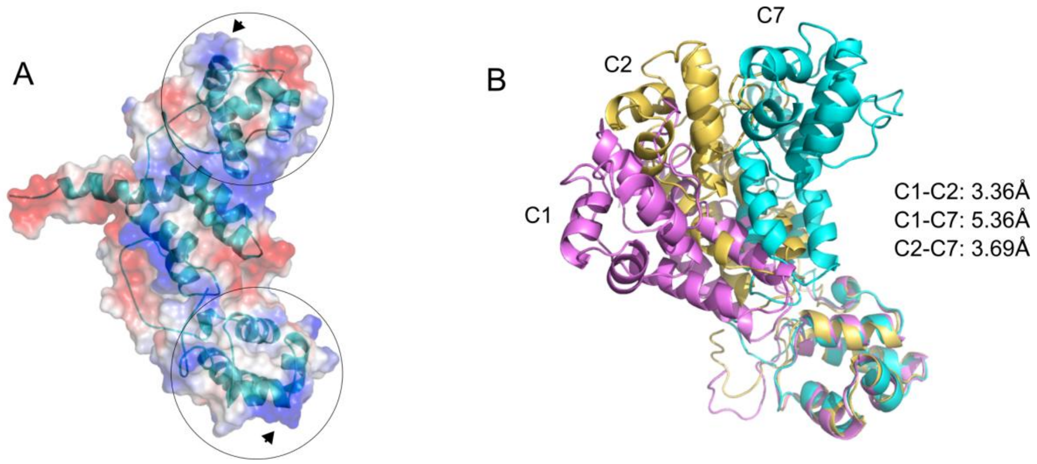

Figure S8: Surface electrostatics and conformational flexibility of *w*Zbi-Wmk. (A) Surface electrostatic potential of the representative *w*Zbi-Wmk conformation (sampled at 85 ns of MD simulation) shown with a cyan ribbon overlay. Surface coloring ranges from red (negatively charged) to blue (positively charged). The circled regions indicate the predicted HTH domains. Black arrows point to the third helix within each domain, which aligns with regions of positive surface potential. (B) Three MD-derived *w*Zbi-Wmk conformations (C1, C2, C7) are anchored at the N-HTH domain to highlight structural flexibility among them. The conformations C1, C2 and C7 were sampled at 25 ns, 35 ns, and 85 ns, respectively. Pairwise structural alignments of the three conformations, measured by root-mean-square-deviation (RMSD), are shown to the right.

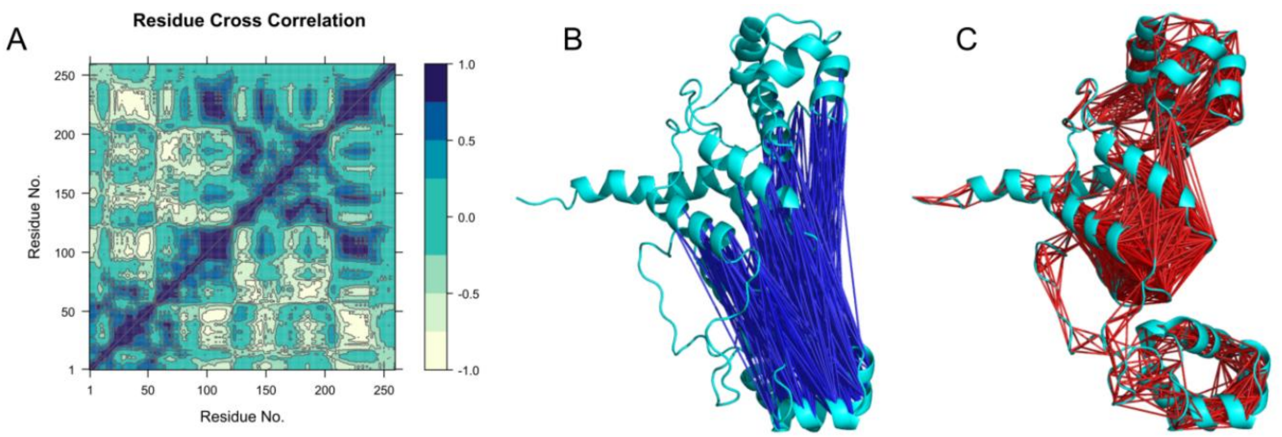

Figure S9: Cross-correlation analysis (CCA) of *w*Zbi-Wmk based on MD simulation. (A) Dynamical cross-correlation map (DCCM) of *w*Zbi-Wmk was derived from MD simulated models after a 20 ns equilibration period. The map shows pairwise atomic displacement correlations across the protein. Positive values indicate correlated motions, whereas negative values represent anti-correlated motions. (B) 3D visualization of dominant anti-correlated motions (correlation values from -1 to -0.8). (C) 3D visualization of dominant correlated motions (values from 0.8 to 1).

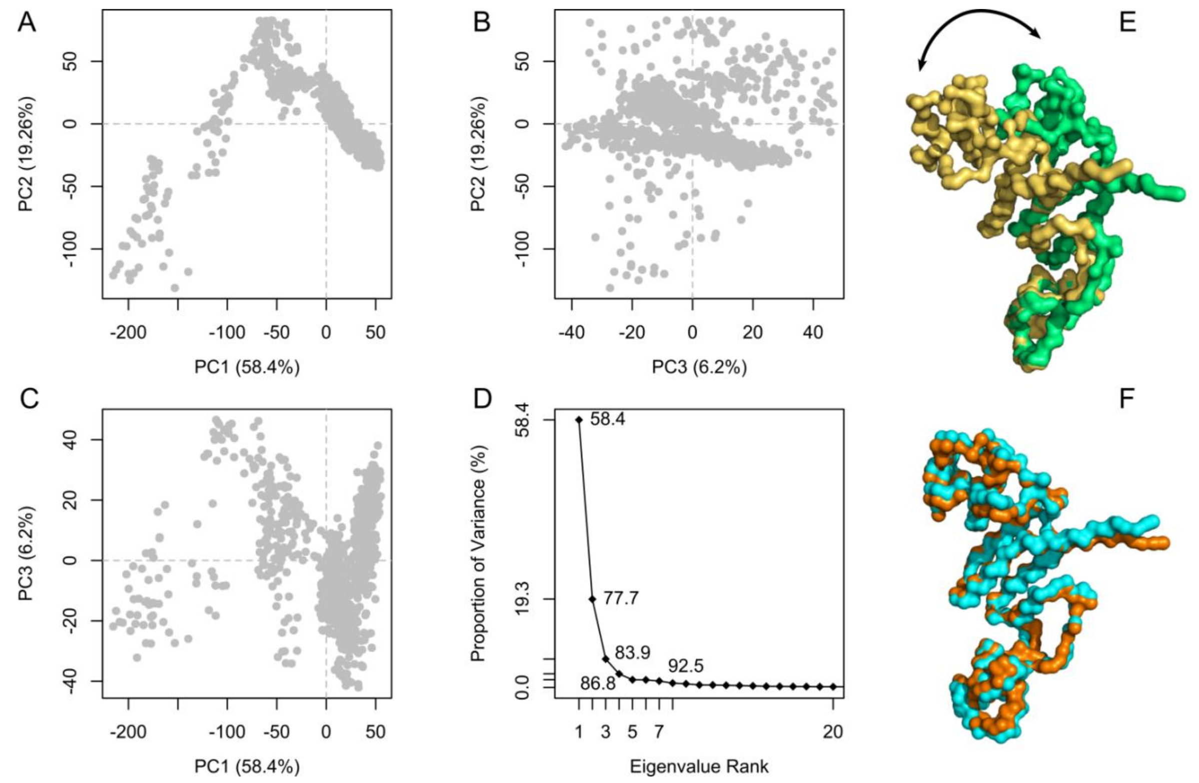

Figure S10: Principal component analysis (PCA) of *w*Zbi-Wmk dynamics from MD simulation. PCA was performed on *w*Zbi-Wmk conformations extracted from the MD-trajectory after a 20 ns equilibration period. (A-C) Scatter plots showing the distribution of conformations projected along the first three principal components (PC1, PC2, and PC3). (D) Scree plot displaying the percentage of total variance explained by each principal components. (E) Structural visualization of major conformational variation along PC2. (F) Structural visualization of major conformational variation along PC3. See Fig. 2 for major changes along PC1.

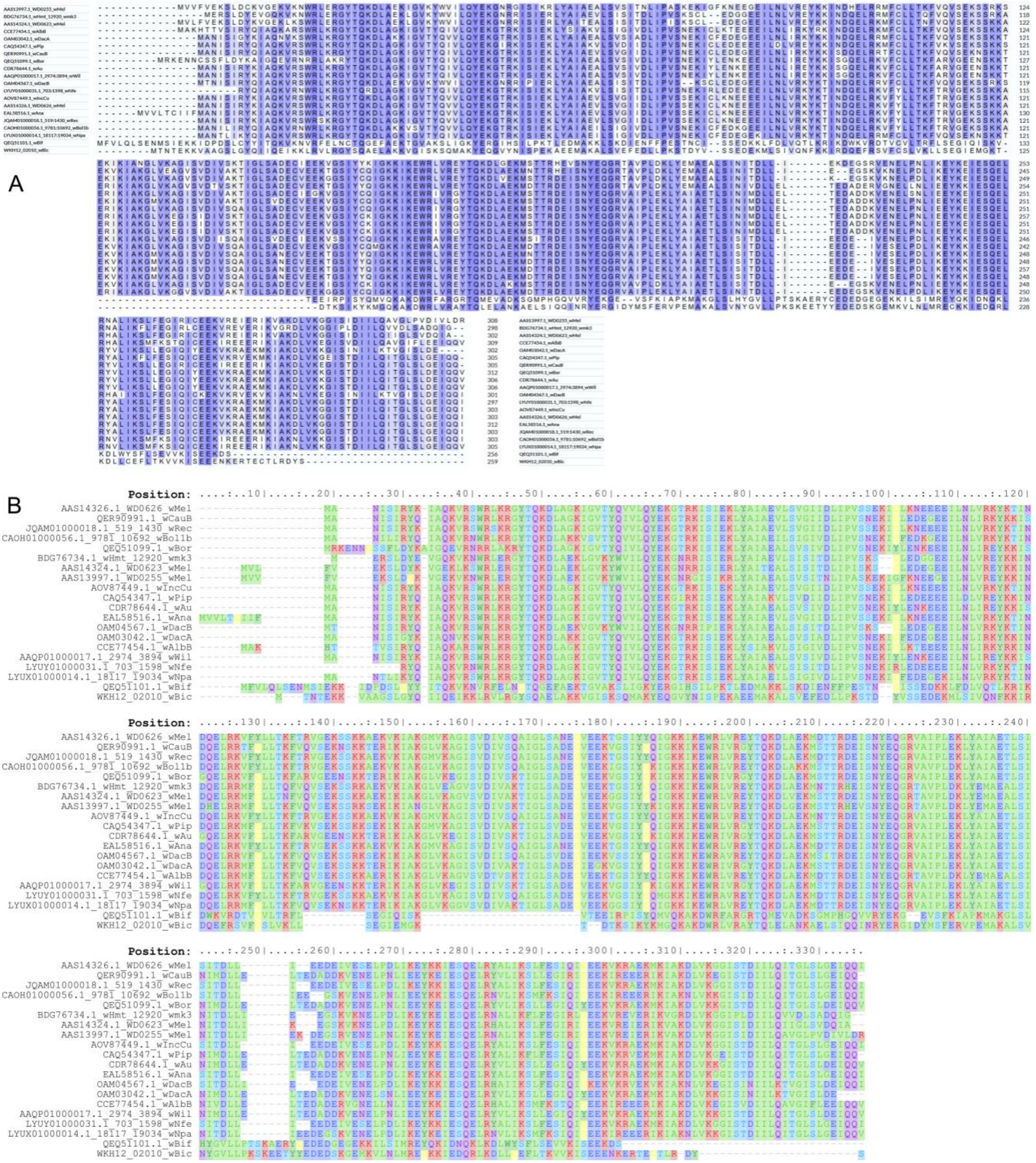

Figure S11: Multiple sequence alignment of Wmk homologs from selected *Wolbachia* strains. Protein sequences of 20 Wmk homologs from *w*Zbi, *w*Bif, *w*Mel, and 15 other *Wolbachia* strains were aligned using two different methods. (A) Alignment generated using Clustal Omega via the Uniprot server. (B) Alignment generated using M-Coffee via the T-Coffee server, which integrates multiple alignment algorithms. The set of homologs used for the alignment is listed in Table S2 (see column 'msa').

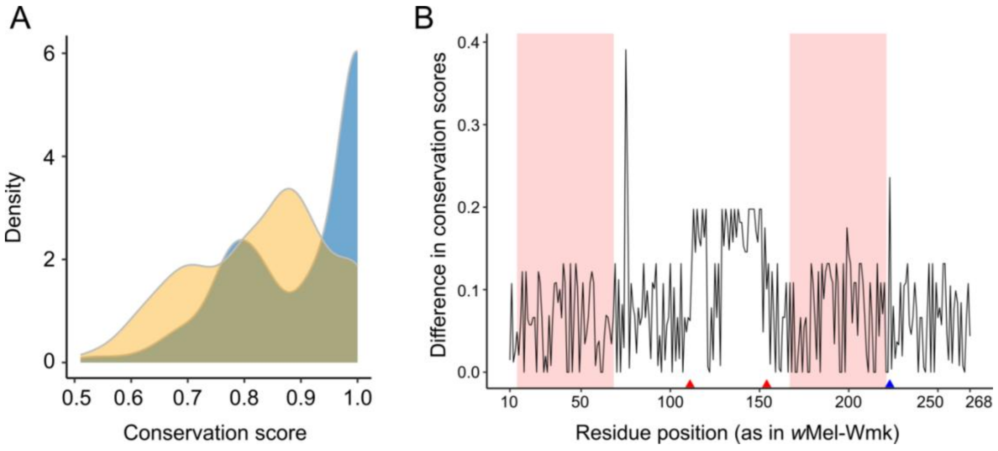

Figure S12: Conservation analysis of Wmk homologs across *Wolbachia* strains. (A) Density plot showing residue-wise conservation scores from multiple sequence alignment. The blue area represents conservation scores derived from alignment excluding *w*Zbi-Wmk and *w*Bif-Wmk, while the orange area includes all of them. Alignments were generated using M-Coffee (T-Coffee web server), and conservation scores were computed for residues 10-268 of *w*Mel-Wmk (see Methods for detail). (B) Plot of the residue-wise difference in conservation scores between the two alignments shown in (A), mapped to *w*Mel-Wmk residue positions.

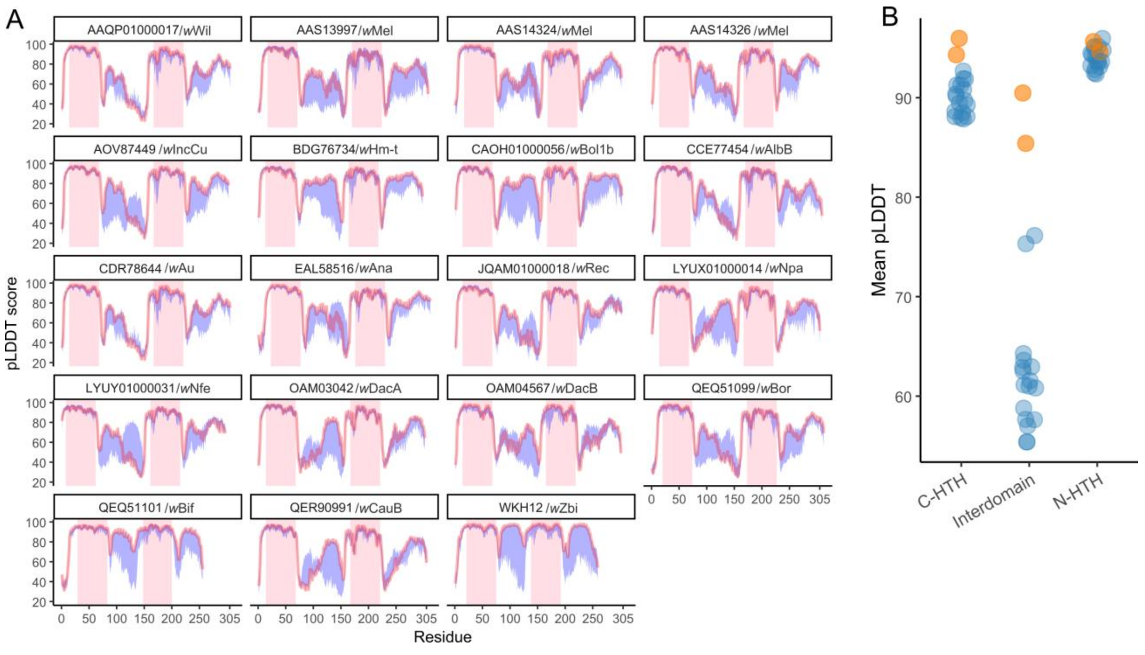

Figure S13: Confidence matrices for AF2-predicted models of Wmk homologs. (A) Residue-wise pLDDT (Predicted local distance difference test) scores of AF2-predicted models (blue line) of 19 Wmk homologs. Structure prediction was unsuccessful for one homolog CAQ54347 from *w*Pip. The blue line represents the pLDDT across five predicted models, while the red line corresponds to the top-ranked model. Predicted HTH domains are highlighted in vertical pink bars. (B) Module-wise mean pLDDT scores across 19 homologs, summarizing structural confidence for each domain or module. Orange circles indicate distant-Wmks (*w*Zbi-Wmk and *w*Bif-Wmk), and lightblue circles, type-I-Wmks. Note: AAS14326: *w*Mel-Wmk, QEQ51101: *w*Bif-Wmk, WKH12: *w*Zbi-Wmk.

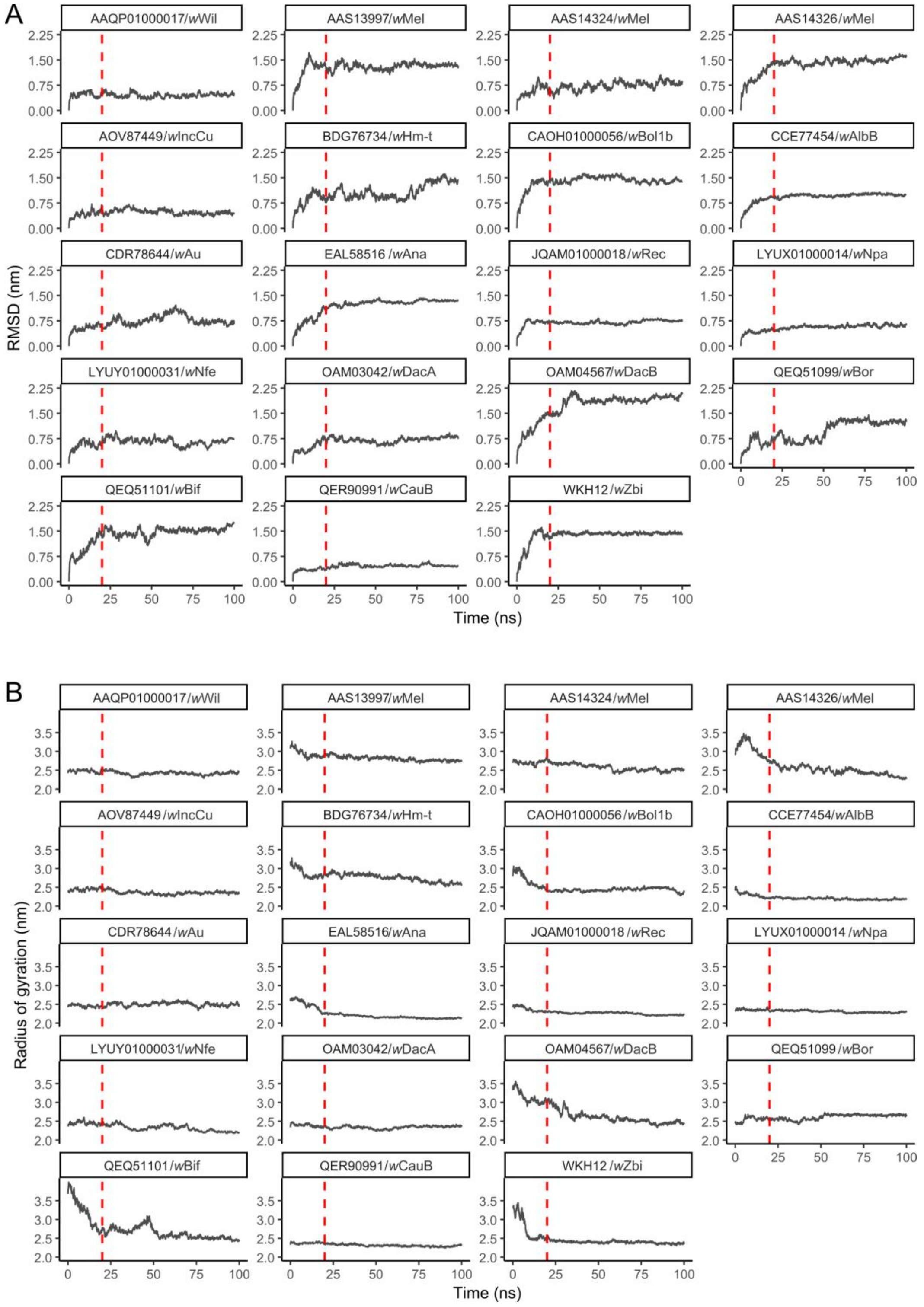

Figure S14: Conformational dynamics of Wmk homologs during MD simulations. Root-mean-square-deviation (RMSD) plots (in A) and radius of gyration (Rg) plots (in B) show changes in overall conformation relative to the initial AF2-predicted structure and the compactness of simulated protein states over time, respectively. Red vertical dashed lines indicate equilibration offset at 20 ns. Note: AAS14326: *w*Mel-Wmk, QEQ51101: *w*Bif-Wmk, WKH12: *w*Zbi-Wmk.

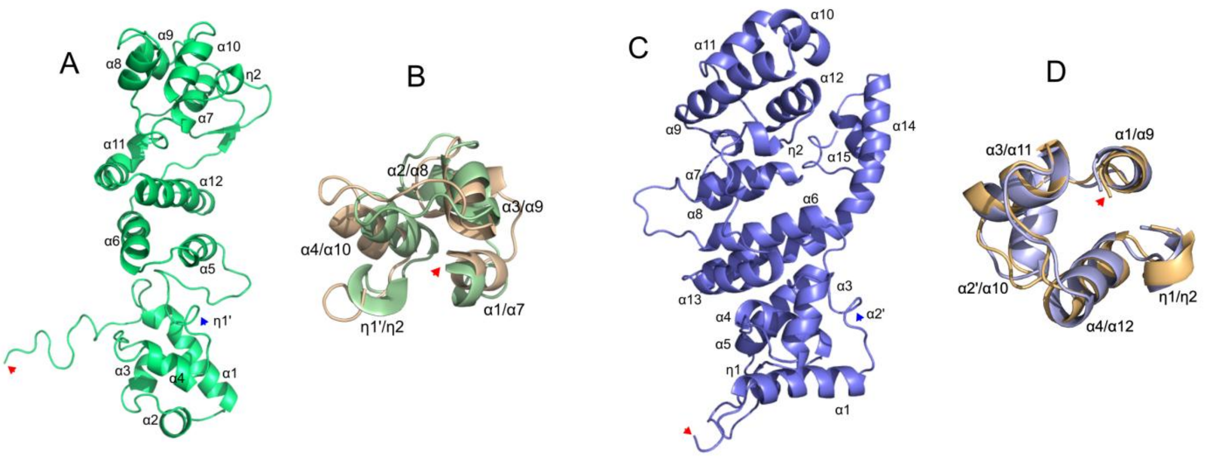

Figure S15: Structural features of *w*Bif-Wmk and *w*Mel-Wmk. (A) Representative conformation of *w*Bif-Wmk retrieved at 85 ns of the MD simulation of the AF2-predicted model. The structure includes 12 complete alpha helices (α1-α12) and two incomplete helices (η1 and η2), likely of a 310-type. The blue mark highlights the absence of η1 (labeled η1') in this conformation compared to original AF2-predicted model (not shown). (B) Structural alignment of N-HTH (wheat brown) and C-HTH (pale green) domains of *w*Bif-Wmk (rmsd=3.173 Å). Overlapped alpha helices are labeled. (C) Representative conformation of *w*Mel-Wmk, comprising 15 alpha helices (α1-α15) and and two incomplete helices (η1 and η2). The blue mark highlights the absence of α2 (labeled α2') in this conformation compared to original AF2-predicted model (not shown). (D) Structural alignment of N-HTH (light orange) and C-HTH (light blue) domains of *w*Mel-Wmk (rmsd=1.465 Å). Overlapped alpha helices are labeled. Across the plots, the red arrows indicate the N-terminus.

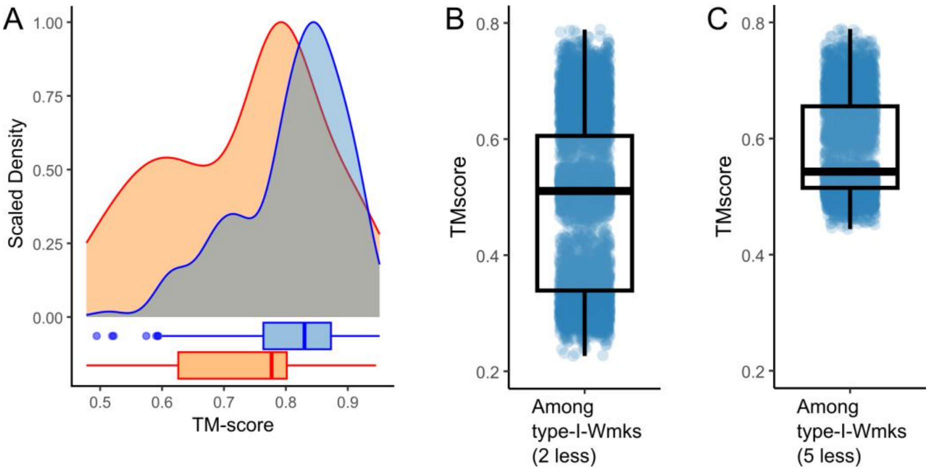

Figure S16: Structural variation among Wmk homologs based on MD-derived conformations. (A) Density plot with overlaid bar charts showing the extent of conformational variation within each homolog. Eight MD-derived conformations per Wmk homolog were compared pairwise using TM-align. Blue indicates type-I-Wmks and orange represents the distant-Wmks. (B) The bar chart with overlaid scatter plot shows pairwise structural alignment of type-I-Wmks, using TM-align, after removing two outlier homologs (*w*Mel-WD0255, *w*DacB-Wmk). (C) The plot shows pairwise structural alignment of type-I-Wmks after removing five outliers (*w*Mel-WD0255, *w*DacB-Wmk, *w*Mel-Wmk, *w*Hmt-Wmk3, *w*Bol1b-Wmk).

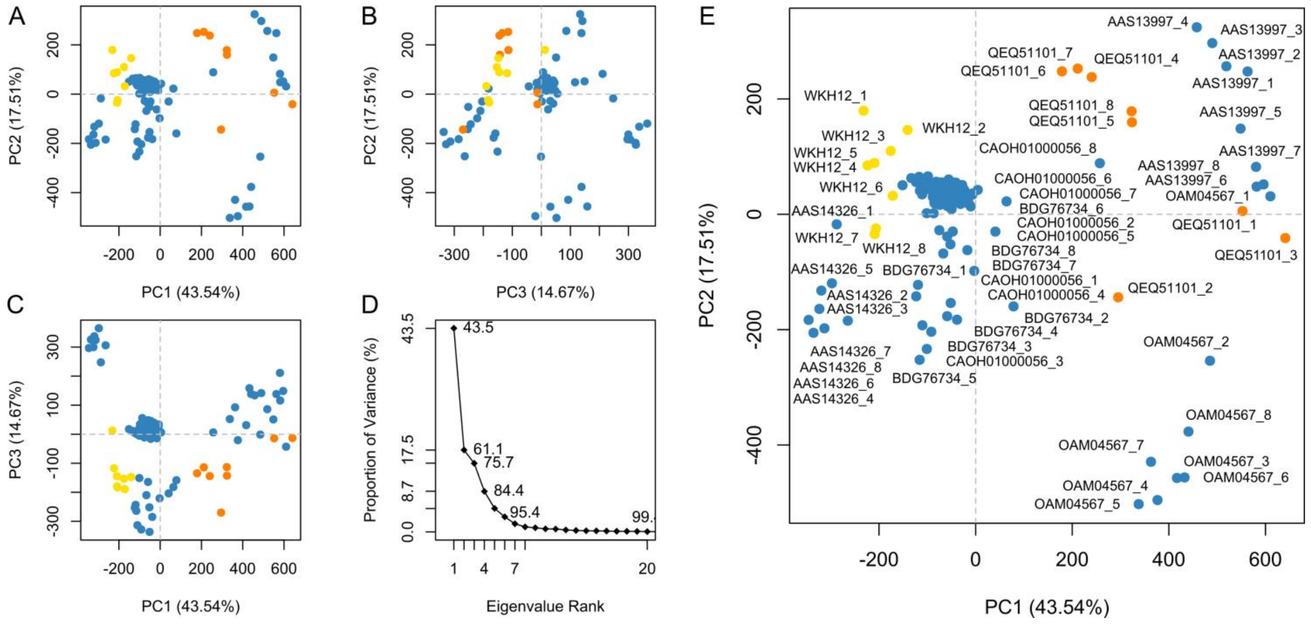

Figure S17: PCA analysis of MD-derived conformations from 19 Wmk homologs. PCA was performed on a combined set of 152 conformations (eight per homolog) obtained from MD simulations of 19 homologs. (A-C) Scatter plots showing the distribution of conformations projected along the first three principal components (PC1, PC2, and PC3). (D) Scree plot displaying the percentage of total variance explained by each principal components. (E) Enlarged view of the PC1-PC2 plot (from panel A), with labeled outlier confirmations. Blue: type-I-Wmks, Orange: *w*Bif-Wmk, Yellow: *w*Zbi-Wmk.

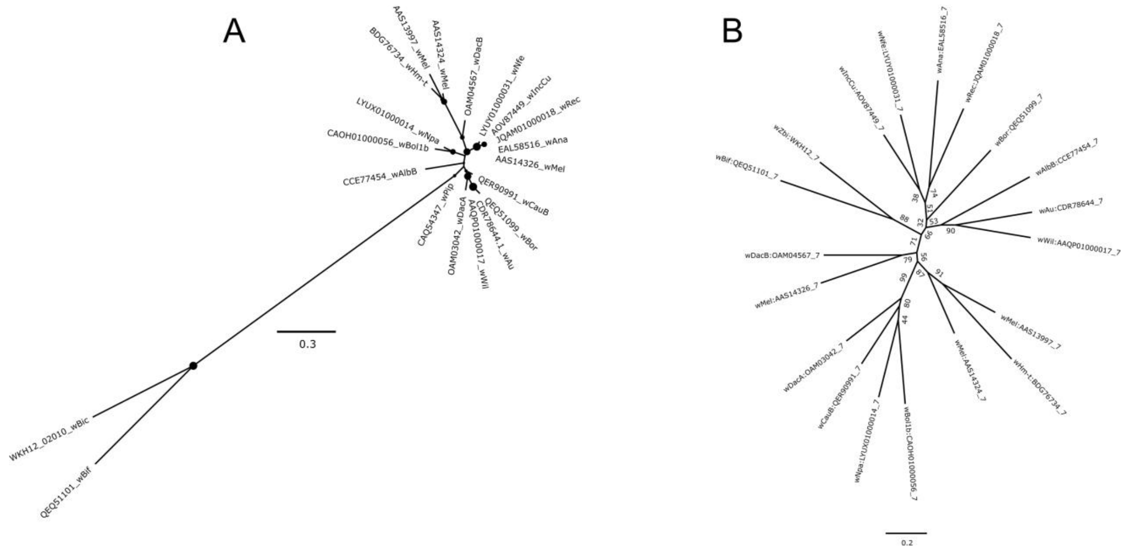

Figure S18: Phylogenetic analysis of Wmk protein from *Wolbachia* strains. (A) Maximum likelihood (ML) unrooted phylogenetic tree reconstructed from amino acid sequence alignment using the best fit model in iqtree. Node support values were estimated from 1000 bootstrap replicates and are shown by varying size of the circles. (B) ML tree from structure-based analysis with node supports from 1000 bootstrap replicates. See Methods for detail. Note: AAS14326: *w*Mel-Wmk, QEQ51101: *w*Bif-Wmk, WKH12: *w*Zbi-Wmk.

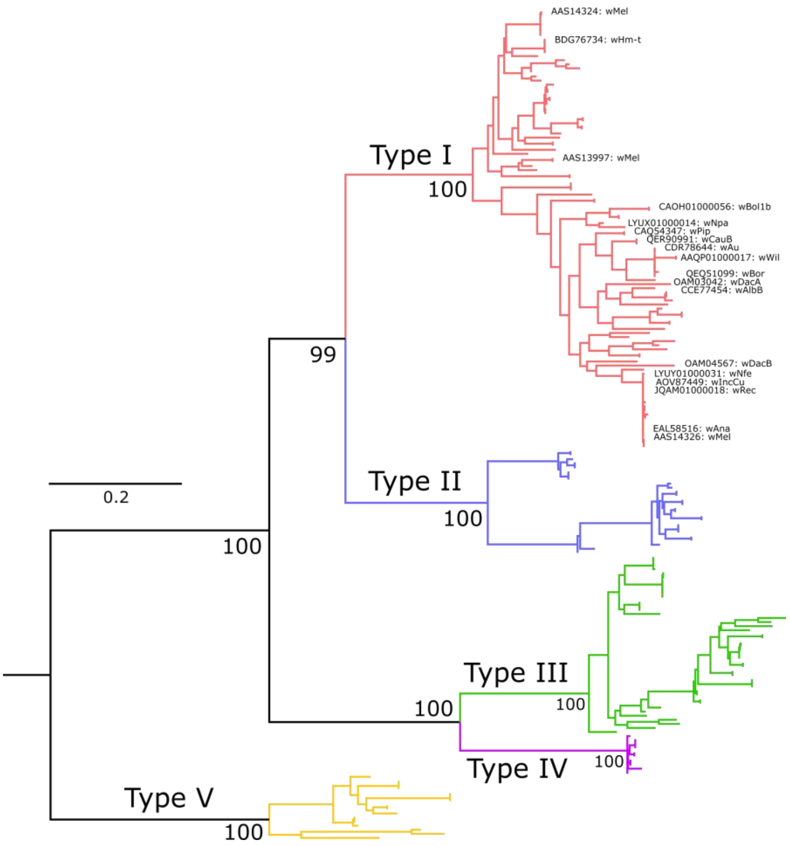

Figure S19: Phylogeny-based typing of *w*Mel-*wmk* and its close homologs in our dataset. This set of homologs were combined with the sequences from Lefoulon et al. (2025), representing all recognized *wmk* types (I–V). Multiple sequence alignment was performed using MAFFT, and a maximum-likelihood phylogeny was inferred using IQ-TREE. Node support values were estimated from 1000 ultrafast bootstrap replicates. The tree is midpoint-rooted between the most divergent groups. Clades are colored according to the established *wmk* typing scheme of Lefoulon et al. (2025). Tip labels are shown only for the homologs from our current dataset.

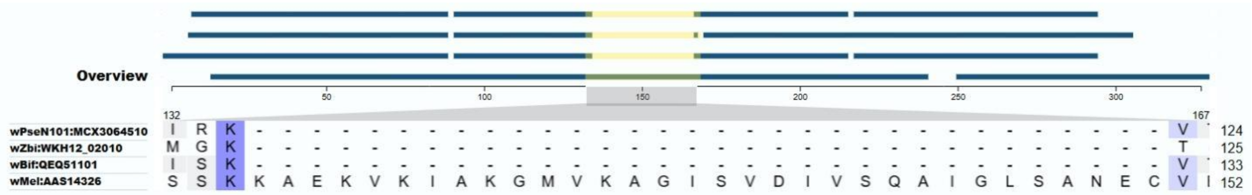

Figure S20: Amino acid sequence alignment of four Wmk homologs: *w*Mel-Wmk, *w*Bif-Wmk, *w*Zbi-Wmk, and the homolog from *w*PseN101. The alignment was generated using Clustal Omega in the UniProt online alignment tool. The top panel provides a graphical overview of the full-length alignment, while the bottom panel shows a zoomed-in view of positions 132–167, highlighting a common sequence deletion present in *w*Bif-Wmk, *w*Zbi-Wmk, and *w*PseN101-MCX3064510 compared to *w*Mel-Wmk.

Video S1: The video shows conformational transition between two MD-derived *w*Zbi-Wmk conformations C1 (start) and C7 (end). The conformations were sampled at 25 ns and 85 ns, respectively, and are anchored at the N-HTH domain (residue 21-75) to highlight the structural flexibility among them, as mentioned in Fig. S8. The intermediate states between the C1 and C7 are predicted using 'morph' function in ChimeraX v1.8.
